# Differential effects of acute, chronic, and social defeat stress on microglial morphology in the basolateral and central amygdala, and the medial and lateral habenula

**DOI:** 10.1007/s00429-026-03172-y

**Published:** 2026-09-04

**Authors:** A. Nieto-Quero, J. Munoz-Martin, M. I. Infantes-López, S. Tabbai, A. Arjona, E. Zambrana-Infantes, P. Chaves-Peña, M. J. Blanca, M. Pérez-Martín, C. Pedraza

**Affiliations:** 1 Departamento de Psicobiología y Metodología de las Ciencias del Comportamiento, Universidad de Málaga, 29010 Malaga, Spain; 2 Departamento de Biología Celular, Genética y Fisiología, Universidad de Málaga, 29010 Malaga, Spain; 3 IBIMA Plataforma BIONAND, Instituto de Investigación Biomédica de Málaga, 29590 Malaga, Spain

**Keywords:** Acute stress, Chronic stress, Social defeat stress, Amygdala, Habenula, Microglia

## Abstract

Effective stress regulation is essential for emotional health, whereas maladaptive stress responses contribute to neuropsychiatric disorders such as depression and post-traumatic stress disorder. Because neuron–microglia interactions are increasingly recognized as key modulators of neuroplasticity under physiological conditions, stress-related microglial dysregulation may contribute to aberrant neuronal responses implicated in mental health disorders. This exploratory study examined region-specific microglial morphometric responses to acute intense stress, chronic restraint stress, and social defeat stress (SDS) in the amygdala and habenula, focusing on the BLA, CeA, LHb, and MHb. Microglial morphology was assessed using animal-level morphometric comparisons and exploratory cluster-based profiling. Acute stress was associated with limited animal-level morphometric differences, while exploratory cluster distributions suggested subregion-dependent shifts, particularly in the CeA and LHb. Chronic stress was associated with larger soma-related parameters in the BLA and CeA, together with a descriptive redistribution toward large-soma/low-process profiles in both amygdalar subregions. In the habenula, chronic stress induced selective MHb morphometric changes, while cluster profiles remained comparatively constrained. SDS was associated with the most pronounced remodelling in the amygdala, including increased soma-related measures, reduced circularity/solidity in the CeA, altered spatial organization, and a strong redistribution toward large-soma/ high-process profile. SDS also shifted LHb cluster profiles, whereas the MHb showed comparatively limited sensitivity. Overall, these exploratory findings suggest that different stressors are associated with region- and subregion-dependent microglial remodelling, with the amygdala showing greater stress-related plasticity than the habenula. These patterns support further investigation of microglial morphological adaptation as a potential contributor to stress-related psychopathology.

**Supplementary Information:**

The online version contains supplementary material available at https://doi.org/10.1007/s00429-026-03172-y.

## Introduction

The ability to cope with stress is crucial for the adaptive success of many species, including humans (McEwen 2007; McEwen et al. 2016). However, when a maladaptive response takes place, stress is a significant risk factor for the development of depression, as well as other neuropsychiatric disorders such as anxiety disorders, posttraumatic stress disorder (PTSD), and schizophrenia, conditions that affect millions of people worldwide (Schneiderman et al. 2005; Charlson et al. 2019; Kupferberg and Hasler 2023; Gebresenbet et al. 2005).

Therefore, it is essential to understand how stress impacts the brain, particularly in structures involved in emotional regulation. In this context, the amygdaloid complex, a critical component of the limbic system, plays an integral role in processing fear and anxiety-inducing stimuli, regulating emotional and behavioural responses, and is also involved in reward mechanisms. Extensive preclinical and clinical research highlights the amygdala as a key mediator in stress responses, significantly influencing brain function and emotional behavior (Chattarji et al. 2015). Within the amygdaloid complex, the basolateral amygdala (BLA) and the central nucleus (CeA) play key roles in integrating emotional behaviours. The BLA regulates emotional responses and processes stress-related stimuli (Maren and Quirk 2004; Ulrich-Lai and Herman 2009), receiving sensory inputs and projecting to various brain regions to shape behavioural and physiological stress responses (Duvarci and Pare 2014). Its dysregulation has been implicated in anxiety and depression, underscoring its role in emotional regulation and stress resilience (Tye et al. 2011; Asim et al. 2024). The CeA is a key output region that orchestrates physiological responses to fear and anxiety, making it crucial for emotional regulation (Yeh et al. 2024). While the BLA processes emotional stimuli, the CeA converts these signals into autonomic and behavioural responses, highlighting their complementary roles in emotional processing (Steinberg et al. 2020; Veinante et al. 2013).

In recent years, the lateral habenula (LHb) has emerged as a key brain region involved in affective regulation and stress-related neuropsychiatric disorders (Jacinto et al. 2017; Baker et al. 2022; Companion et al. 2022; Piper et al. 2024; Proulx et al. 2014). The LHb regulates avoidance and anti-reward behaviours, contributing to its emerging significance in depression. Elevated LHb activity has been observed in various animal models of depression, with studies showing that such hyperactivity can be reversed by antidepressant treatments or deep brain stimulation, thereby reducing depressive symptoms. Beyond depression, aberrant LHb function has also been implicated in a broad range of neuropsychiatric conditions, positioning it as a critical hub within the neural circuitry underlying these disorders (Hu et al. 2020; Baker et al. 2022).

Along with the LHb, the medial habenula (MHb) has also shown significant involvement in modulating stress responses, albeit to a more moderate degree. Lesion studies targeting the habenula and transections of its efferent pathways in the forebrain have demonstrated pronounced increases in both endocrine and behavioural indices of stress (Lee and Huang 1988; Murphy et al. 1996; Thornton et al. 1994). The MHb is involved in regulating higher brain functions, including reward, attention, mood, and decision-making (Lee and Goto 2011; Fowler et al. 2013; Kobayashi et al. 2013). Dysfunction of the MHb has been linked to psychiatric diseases such as anxiety and anhedonia as a core symptom in depression (Hsu et al. 2014; Lecourtier et al. 2004; Lee and Goto 2011; Kobayashi et al. 2013; Yamaguchi et al. 2013).

Recent evidence suggests that neuron-microglia interactions are crucial for modulating neuroplasticity, neural circuit function, and behaviour under physiological and pathological conditions (Hartmann et al. 2024; Zhao et al. 2024). Microglia, the resident immune cells of the central nervous system, are essential for maintaining homeostasis and responding to pathological insults. They exhibit remarkable plasticity, adapting their morphology and function in response to environmental cues. Under stress conditions, microglia can react, shifting to a pro-inflammatory state that can influence neuronal function and behaviour (O’Connor and Nissen 2023; Pallarés-Moratalla and Bergers 2024). Dysregulation of microglia, particularly through an increased microglial response to stress, may lead to aberrant neuronal responses, implicating microglia in the neurobiology of mental health disorders (Kreisel et al. 2014; Wohleb 2016). Stress models in rodents have shown neuronal dystrophy accompanied by alterations in microglia morphology, which can impact their function and suggest a state of inflammation. While this activation has been well-documented in the hippocampus (Nieto-Quero et al. 2021; 2023; Tay et al. 2018; White et al. 2024), the effects of stress-induced microglial alterations in the amygdala and habenula remain poorly understood. Given the critical roles of these regions in emotion and behaviour, understanding microglial changes in these areas is vital for comprehending the full impact of stress on the brain. Therefore, the primary aim of this study was to investigate microglial responses in the amygdala (BLA and CeA) and the habenula (LHb and MHb) in response to different stressors (acute intense stress as an animal model of post-traumatic stress disorder, chronic moderate stress, and chronic social defeat stress).

## Material and methods

### Animals

All animal procedures included in this project were carried out in compliance with the European regulations for animal research (General Rules from the European Community Council 2010/63/UE, 90/219/CEE, Regulation (EC) No. 1946/2003) and Spanish National Guidelines for Animal Experimenting (Real Decreto 53/2013), and were approved by the University of Malaga Ethical Committee (CEUMA 51–2018-A; CEUMA 2–2019-A) and corroborated by the Agriculture, Farming, Fishing and Sustainable Development Council (10/06/2019/114).

In this study, 24 male C57BL/6 J mice (Janvier Laboratories, Le Genest-Saint-Isle, France), all aged two months at the onset of the experiment, were employed. Each mouse was individually housed for one week prior to the initiation of the stress protocols under standardized conditions (temperature: 22 ± 2 °C; humidity: 55 ± 5%; 12-h light/dark cycle) with ad libitum access to food and water. The experimental design included three distinct stress-inducing protocols to model various stress responses: acute stress as a model for PTSD, chronic and moderate stress, and chronic social defeat stress. All procedures were conducted daily between 08:00 and 15:00 h. To ensure experimental rigor and minimize biological and technical variability, each experimental group was processed and analysed concurrently with its own dedicated control group. These independent sets of experiments were conducted at different time points to prevent inter-experimental interference and to account for potential cohort or litter-specific differences. Accordingly, procedural controls were tailored to each paradigm; for the chronic stress cohort, this included vehicle (VEH) administration for both stressed and control animals to ensure baseline consistency during microglial assessment.

An additional cohort of mice was incorporated into the social defeat protocol, although morphometric changes in microglia were not the focus of this study. This cohort comprised five male OF-1 mice (Charles River Laboratories France, Inc., Lyon, France), each aged three months at the commencement of the study. These subjects were individually housed in cages measuring 15.5 × 31.5 × 14 cm, starting 12 days prior to the initial social defeat session. Housing was maintained throughout the experiment's duration. Cage maintenance was conducted on a weekly basis prior to initiating the stress protocol; however, this routine was suspended during the protocol to promote the accumulation of odorant markers and enhancement of territorial behaviours. To augment aggressiveness, opaque white barriers were installed between the cages to obstruct visual contact among the mice, effective immediately upon their segregation.

## Stress procedures: experimental groups

All procedures were conducted in a specialized room where animals were acclimated at least 45 min before the start of the day's protocol. This structured approach ensures rigorous examination of stress impacts across different modalities, enhancing the understanding of stress-related morphological and behavioural changes*.*

### Acute stress protocol

For the acute stress induction, reminiscent of PTSD models, subjects were exposed to water immersion restraint stress (WIRS). Mice were immobilized in falcon tubes, measuring 30 mm in diameter × 80 mm in length) and submerged to the level of the xiphoid process in a water bath maintained at 23 ± 1 °C for 2 h (Ohgidani et al. 2016; Nieto-Quero et al. 2023; Infantes-López et al. 2023). Two experimental groups were established: Control (n = 4) and acute WIRS (n = 4). Control animals remained in standard housing conditions on a 12-h light/dark cycle, with ad libitum access to food and water throughout the stress application period, except during behavioural tests modelling depression.

### Chronic stress protocol

Chronic stress was carried out using clear polystyrene conical centrifuge tubes, modified with air holes for ventilation and with a diameter of 50 mm. This immobilization protocol, aimed at increasing unpredictability, was implemented intermittently (5 days of stress application followed by 2 days of rest, and then another 5 days of stress). Each session varied in start times (between 08:00 and 12:00) and durations (ranging from 3.5 to 5.5 h per day). Two experimental groups were established: Control (n = 4) and chronic restraint stress (n = 4). This protocol has been effectively implemented and is extensively detailed in our group's prior studies, demonstrating its utility in modelling chronic stress (Moreno-Fernández et al. 2020). Control groups, also consisting of four animals each, were maintained under standard conditions with a consistent 12-h light/dark cycle*.*

### Social defeat stress protocol

Two experimental groups were established: Control (n = 4) and Social Defeat Stressed animals (SDS) (n = 4). On each day of the 10-day protocol, mice underwent a 25-min session consisting of three consecutive phases. First, the intruder mouse was placed in the resident mouse’s home cage for 10 min behind a transparent methacrylate divider, which allowed visual, olfactory, and auditory contact while preventing physical interaction. The divider was then removed for 5 min to permit direct interaction and subsequently reinserted for the final 10 min, during which sensory contact was maintained without physical contact. To qualify resident OF-1 mice as sufficiently aggressive for the SDS protocol, a pre-screening using the Golden et al. (2011) method was employed, involving potential combativeness demonstrated within a three-minute window over consecutive days.

### Histology

#### Tissue processing and immunohistochemistry

Following the completion of the stress protocols, mice were anaesthetized with 200 mg/mL sodium pentobarbital and intracardially perfused with PBS (pH 7.4) and 4% paraformaldehyde and post-fixed for 24 h in paraformaldehyde. The brains were post-fixed and cut into six series of coronal Sects. (40 µm) using a vibratome (Leica VT1000S, Leica Biosystems) and stored in six series. Thus, each section was 240 μm apart from the next section within each series for the histological analysis. Sections were stored in cryoprotectant solution (30% ethylene glycol, 30% sucrose) at − 18 °C until further use.

To identify microglia a rabbit anti-Iba1 (1:500; Wako, ref: 019–19741) was used as primary antibodies. Then, we used anti-rabbit (1:1000; Dako, ref: E0432) as biotinylated secondary antibodies. See Nieto-Quero et al. (2023) for details.

The presence of ionized calcium-binding adapter molecule 1 (Iba-1), a specific marker for microglia, was assessed via indirect immunocytochemistry. Tissue sections were treated with a biotin-conjugated anti-Iba-1 antibody linked to peroxidase. Following the development phase, the staining complex precipitated, marking the microglia. The sections were then mounted and subsequently dehydrated.

#### Image acquisition and microglial morphometric analysis

**Fig. 1:**
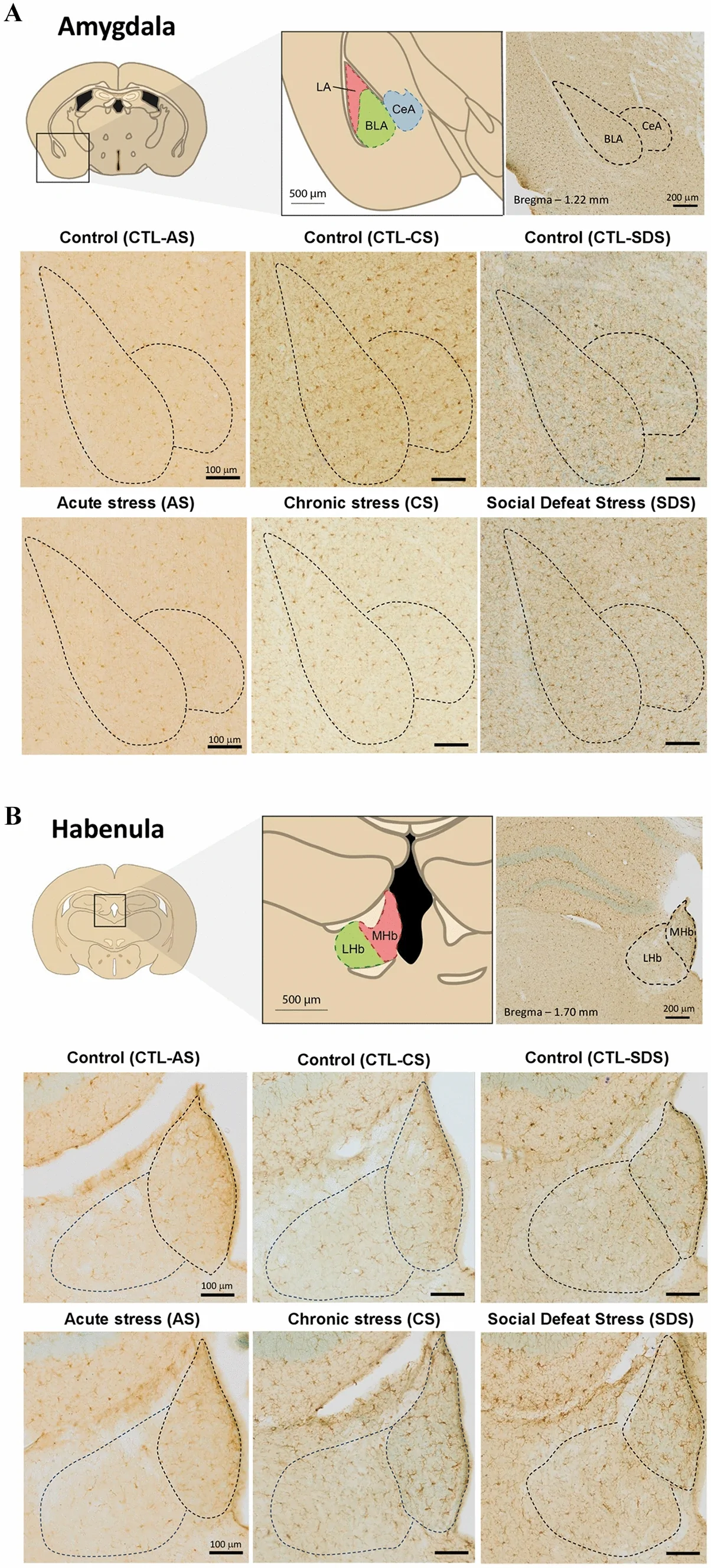
Representative photomicrographs of Iba1-immunoreactive microglia in the amygdala (basolateral amygdala, BLA, and central amygdala, CeA; upper panels, **A**) and the habenula (medial habenula, MHb, and lateral habenula, LHb; lower panels, **B**) under different experimental conditions. For each stress paradigm, images from stressed animals are shown alongside their respective control cohort. Low-magnification images provide an overview of each region at standardized rostrocaudal levels (Bregma − 1.22 mm, − 1.46 mm, and − 1.70 mm; scale bar = 100 µm), while high-magnification insets illustrate microglial morphology in greater detail (scale bar = 200 µm)

Tissue sections immunostained with anti-Iba1 were scanned at 20 × magnification using an Olympus VS120-5S virtual microscope and VS-ASW software (Olympus, Tokyo, Japan). For each section, a 10-µm z-range was scanned, and the resulting z-stack was combined into a single RGB image (32 bits per pixel) saved in TIFF format, with a pixel size of 0.3450 × 0.3450 µm per pixel and a resolution of 2.8986 pixels per µm. Image analysis was subsequently conducted using Fiji/ImageJ software (Schneider et al. 2012;), applying the analytical procedures described by Davis et al. (2017) and following previously published protocols (Nieto-Quero et al. 2023; Infantes-López et al. 2023). A total of four animals per experimental condition were used. For all univariate statistical analyses, section-level measurements were first averaged within each animal, and the animal was used as the biological unit of replication. Image acquisition and ROI delineation were performed blind to experimental condition. Moreover, subsequent morphometric quantification was conducted using fixed automated ImageJ/Fiji macros and predefined segmentation criteria applied uniformly across all images, thereby reducing operator-dependent variability during image processing and quantification. Regions of interest (ROIs) were identified on the 20 × scanned images and manually delineated based on the neuroanatomical references of Paxinos & Franklin (2004), using Bregma coordinates matched to the corresponding histological sections. Specifically, microglial morphology was assessed in the basolateral amygdala (BLA), central amygdala (CeA), medial habenula (MHb), and lateral habenula (LHb). For the amygdala, 3–9 coronal sections per animal were analysed (Bregma – 0.58 mm to – 2.30 mm), whereas for the habenula, 2–5 sections per animal were analysed (Bregma – 0.94 mm to – 2.18 mm). The number of quantified microglial somas varied according to brain region, subregion, and experimental condition. Mean values per animal were consistently higher in the BLA than in the CeA, ranging approximately from 106 to 221 microglial somas per animal in the BLA and from 43 to 67 in the CeA. In the habenula, mean values also differed between subregions, ranging approximately from 7 to 29 microglial somas per animal in the MHb and from 12 to 22 in the LHb. Representative photomicrographs for each region and experimental condition are shown in Fig. 1 (amygdala, Fig. 1A; habenula, Fig. 1B).

#### Microglial soma analysis

**Fig. 2:**
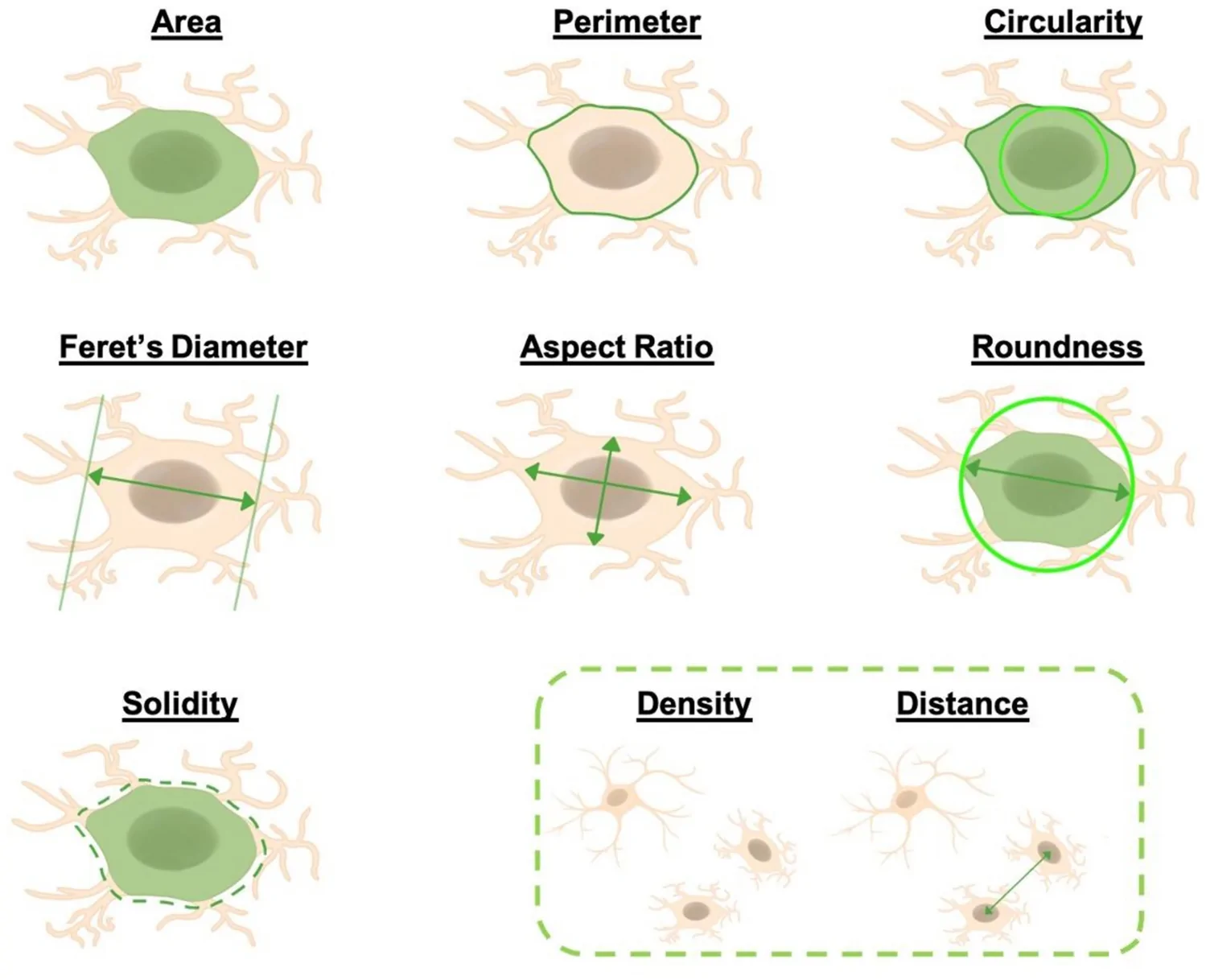
Schematic overview of the microglial soma morphometric analysis. The analysis includes quantitative measurements of soma size (e.g., area and perimeter), shape descriptors (e.g., circularity, roundness, aspect ratio, and Feret diameter), and spatial distribution parameters used to characterize stress-induced morphological changes

Microglial soma isolation was performed using an ImageJ/Fiji macro optimized by the Image Analysis Service of the Central Information Technology Service (SCI) at the University of Málaga (UMA). The macro was applied uniformly across all images to extract standardized morphometric descriptors of Iba1-positive microglial somas (Fig. S1A and S1B). The complete macro code is provided in the Supplementary Methods section under “Macro code for microglial morphometry”. For each section, regions of interest (ROIs) corresponding to the BLA, CeA, LHb, and MHb were delineated using consistent anatomical criteria across all animals and experimental groups. The selected ROIs were then subjected to morphometric quantification using fixed automated ImageJ/Fiji macros and predefined segmentation criteria applied uniformly across all images, thereby reducing operator-dependent variability during image processing and quantification. Briefly, images were converted to 8-bit grayscale, corresponding to a 0–255 intensity range, and background subtraction was performed using a rolling ball radius of 50 pixels. Images were then binarized using a black background,and a fixed threshold value of 180 was applied to segment Iba1-positive microglial somas. Morphological closing and opening operations were subsequently used to improve particle segmentation before particle analysis. Finally, a dual-filtering approach was applied to restrict Iba1 staining exclusively to cell bodies and eliminate processes: first, the aforementioned grayscale threshold was utilized, followed by a size particle filter to include only areas between 15 and 200 px^2^. In total, nine quantitative parameters were assessed to characterize soma size (S), morphology (M), and distribution (D) (see Fig. 2):

*Soma Area* (S): Measures the surface area occupied by each microglial cell soma, reported in μm^2^.

*Perimeter* (S): Determines the contour length of each cell soma, reported in μm.

*Circularity* (M): Quantified by Eq. (1), Its value lies in the interval [0, 1], where values approaching 1 indicate near-perfect circularity, suggesting a rounded shape, whereas values closer to 0 indicate an elongated shape.

1 $$\mathrm{Circularity}=\frac{4\pi \times (\mathrm{Soma} \mathrm{area}) }{\text{(Perimeter) 2}}$$

*Feret Diameter* (M): Represents the longest distance between any two points along the perimeter of the microglial soma, with larger values indicating more elongated shapes, reported in μm.

*Aspect Ratio* (M): Defined by Eq. (2), this metric evaluates the ratio of the major axis to the minor axis within the cell soma, providing insight into the ellipticity of the soma.

2 $$\text{Aspect Ratio} (\mathrm{AR})=\frac{\text{Major axis length} }{{\rm Minor\, axis\, length}}$$

*Roundness* (M): Measures the homogeneity of the cell soma's distribution, calculated as the circularity corrected by the aspect ratio, described in Eq. (3).

3 $$\mathrm{Roundness} = \frac{4 \times (\mathrm{Soma} \mathrm{area}) }{\pi \times \left(\text{Major axis length}\right)2}=\frac{1}{AR} $$

*Solidity* (M): Assesses the pixel density of the cell soma, as calculated by the ratio of the soma area to the convex area—the latter being the smallest polygon that can enclose the soma while maintaining convexity (all internal angles less than 180°). Higher solidity values suggest a shape closer to a perfect polygon, as detailed in Eq. (4).

4 $$\mathrm{Solidity} =\frac{ \text{Soma area}}{\text{convex area}}$$

*Density* (D): Number of microglial somas within the defined tissue region, calculated as the total count of microglia divided by the area of the Region of Interest (ROI) and expressed as soma per µm^2^.

*Distance* (D): Distance between each analysed microglial soma and its closest neighbouring soma, calculated using the centroid of each cell body as the reference point and expressed in µm.

This comprehensive analysis offers critical insights into the microglial morphology and spatial distribution, facilitating a deeper understanding of the cellular responses under various experimental conditions.

#### Microglial processes analysis

Microglial processes analysis was conducted as previously described (Infantes-López et al. 2023). The same original 20 × scanned Iba1-immunostained sections and the same Regions of Interest (ROIs) used for soma analysis were analysed, ensuring direct comparability across measurements (N = 4 animals per experimental condition).

An established ImageJ/Fiji macro procedure was used to quantify threshold-dependent Iba1-positive area coverage within each ROI as an indirect morphometric descriptor related to microglial process signal (Supplementary Fig. S1C). The macro code for this procedure is publicly available in GitHub (https://github.com/Jorvalgl/Thresholderer). In brief, images were automatically converted to 8-bit grayscale, background-subtracted using a rolling ball radius of 50 pixels and subjected to sequential binary thresholding across the full grayscale range (0–255; black background). For each threshold value, the total pixel area occupied by Iba1-positive signal within the ROI was quantified. Because this measure captures the overall Iba1-positive area, it includes signal from both microglial somas and processes. The percentage of Iba1-positive area relative to the total ROI area was calculated for each threshold value, and these values were plotted to generate a threshold-dependent curve (Supplementary Figs. S2 and S3).

To estimate the contribution of microglial processes in these binary images, a two-step mathematical procedure was used. First, the percentage of Iba1-positive area associated with somas within the ROI was calculated per section and averaged per animal by multiplying the mean soma area by the mean number of somas within the analysed region, as derived from soma analysis, and normalizing this value to the total ROI area. Second, these soma-associated values were subtracted from the total Iba1-positive area measured at each threshold, as described in the following equation:

5 $$ \begin{aligned} \% \mathrm{Processes} & = \frac{\mathrm{Area(Processes)}}{\text{Area (ROI)}}\\ & =\frac{\text{Area (Total Iba1staining)}}{\text{Area (ROI)}} \\ & \quad -\frac{\mathrm{Area}\left(\mathrm{soma}\right) \times \text{Number (somas)}}{\mathrm{Area(ROI)}} \end{aligned}$$

This yielded a threshold-dependent curve of the percentage of Iba1-positive area associated exclusively with microglial processes within the ROI (Supplementary Figs. S5 and S6).

Threshold-range selection was based exclusively on control sections and was subsequently held constant for all animals within each analysis. Specifically, a control-calibrated sensitivity analysis was performed (Supplementary Fig. S4), in which control sections from each stress paradigm and subregion were binarized across consecutive threshold values in steps of 10 grayscale units in order to identify a stable interval in which thin Iba1-positive processes were consistently preserved while minimizing background inclusion and soma over-segmentation. On the basis of this calibration, the threshold range of 25–45 grayscale units were selected to represent microglial processes. For the main analyses, the percentage of Iba1-positive area associated with processes was averaged across this fixed threshold interval for each section and then per animal. This approach reduced dependence on a single threshold value while keeping threshold selection independent of between-group comparisons. Threshold-sweep plots were retained as exploratory sensitivity analyses to document the stability of signal detection across neighboring grayscale thresholds and were not used to select thresholds based on group differences. Representative binary masks illustrating segmentation quality within the selected threshold interval are provided in the Figure S4. Because each stress paradigm used an independent control cohort with distinct baseline intensities, absolute inter-paradigm comparisons should be interpreted with caution.

### Statistical analysis

We performed two-tailed independent-samples t-tests or Welch’s t-tests, as appropriate, to assess differences in microglial parameters between control and experimental groups across the different stress-related paradigms. Adusah and Brooks (2011) showed that the *t*-test controls for type I error better than Welch's *t*-test when the assumption of homogeneity of variance is met and the sample size is very small. However, they found that Welch's *t*-test was superior to the *t*-test when this assumption is not met and that pre-testing for equality of variance with Levene's test did not provide advantages over using Welch's *t*-test by default. Consequently, we performed Welch's *t*-test when the standard deviation was not equal in the two groups. The variables analysed included soma morphometric measures (area, perimeter, circularity, Feret diameter, aspect ratio, roundness, and solidity), spatial distribution metrics (nearest-neighbor distance and density), and the percentage of microglial processes. Analyses were conducted separately within defined subregions of the amygdala (basolateral amygdala, BLA; central amygdala, CeA) and the habenula (medial habenula, MHb; lateral habenula, LHb). For all univariate analyses, the animal was used as the biological unit of replication (n = 4 animals per group within each paradigm). Section-level values were first averaged within each animal, and group comparisons were subsequently performed on animal-level means.

Given the exploratory, regionally resolved nature of the present morphometric analysis, the modest sample size, and the partial interdependence among several endpoints reflecting related aspects of soma size, shape, and spatial organization, no formal multiplicity correction was applied as the primary inferential criterion. We acknowledge that the number of comparisons increases the risk of type I error, whereas the limited number of animals per group increases the risk of type II error. Accordingly, p-values are reported as nominal and unadjusted, and results were interpreted cautiously alongside effect sizes, 95% confidence intervals, and convergent patterns across related morphometric parameters. The findings are therefore presented as exploratory. For each comparison, effect size was estimated using Hedges’s g, which corrects for small-sample bias (Hedges and Olkin 1985). Exact two-tailed p-values, degrees of freedom, Hedges’s g, and 95% confidence intervals for the mean difference are reported in the tables. Following the guidelines proposed by Cohen (1988), effect sizes of 0.2, 0.5, and 0.8 were respectively interpreted as small, medium, and large.

Statistical analyses were conducted using SPSS version 28 and JASP Team (2023).

### K-means clustering

To classify microglial morphometric profiles, soma area and process coverage were selected as the two clustering inputs, as they represent complementary soma- and process-related morphometric descriptors. Both variables were z-score standardized before clustering. Circularity and density were standardized separately and used only as additional morphometric descriptors to characterize the resulting clusters; they were not included as clustering inputs.

K-means clustering was applied to section-level observations to preserve local anatomical heterogeneity within each subregion. The algorithm partitions observations by minimizing the total within-cluster sum of squared Euclidean distances to the corresponding cluster centroids. For each analysis, the algorithm was initialized with 10 random starting configurations (nstart = 10), and the solution with the lowest total within-cluster sum of squares was retained. A fixed random seed (set.seed(123)) was applied to ensure reproducibility. All analyses were conducted in R version 4.4.3 (2025–02-28, “Trophy Case”) using the stats::kmeans() function. Artificial intelligence tools were used to optimize R scripts for cluster analysis. All analyses were performed and verified by the authors, with selected clusters cross-checked in jamovi (version 2.3.18.0). 

Because sections were nested within animals, cluster assignments were subsequently aggregated at the animal level before group-level summaries and statistical comparisons. For each animal, the proportion of sections assigned to each cluster was calculated, and group-level values were expressed as the mean ± SD of the four animal-level proportions. Section-level counts and animal-level cluster proportions are reported in Table 7 and Supplementary Material, together with the number of sections and microglia contributing to each analysis.

For each subregion and stress paradigm (BLA, CeA, MHb, and LHb under acute stress, chronic stress, and SDS conditions) a K = 2 solution was selected as an exploratory approach. The quality of the clustering solution was assessed using internal validation indices, including the Silhouette index, Calinski–Harabasz index, Davies–Bouldin index, and BetweenSS/TotalSS. Cluster centroids and standardization details are reported in Supplementary Table S1 to allow reproducibility of the cluster solutions. Because cluster solutions were generated separately for each subregion and stress paradigm, cluster labels and centroid values were interpreted only within each specific analysis and were not compared directly across paradigms. In addition, because each stress paradigm used an independently processed control cohort, absolute morphometric values or cluster distributions were not interpreted as baseline differences across paradigms. To examine whether the resulting clusters differed in morphometric variables not used for clustering, circularity and density were compared between clusters using two-tailed Student’s t-tests or Welch’s t-tests, as appropriate. Effect sizes were estimated using Hedges’ g. These comparisons were considered exploratory morphometric characterizations of the clusters rather than independent biological validation. Throughout the manuscript, clusters are described as exploratory morphometric profiles rather than discrete biological or functional microglial states.

To assess the stability of the exploratory k-means partitions, animal-level bootstrap resampling was performed. For each dataset, 100 bootstrap iterations were conducted. In each iteration, animals were sampled with replacement, and all sections belonging to each selected animal were retained. K-means clustering was then repeated using the same settings as in the original analysis (K = 2, nstart = 10). Bootstrap-derived clustering solutions were projected onto the original dataset and compared with the original k-means solution using the Adjusted Rand Index (ARI) and cluster-wise Jaccard similarity. Bootstrap stability metrics are reported in Supplementary Table S2. These analyses were used to assess the stability of the exploratory morphometric partitions under animal-level resampling and were not interpreted as independent biological validation of discrete microglial states.

## Results

### Acute stress (PTSD model) analysis

**Fig. 3:**
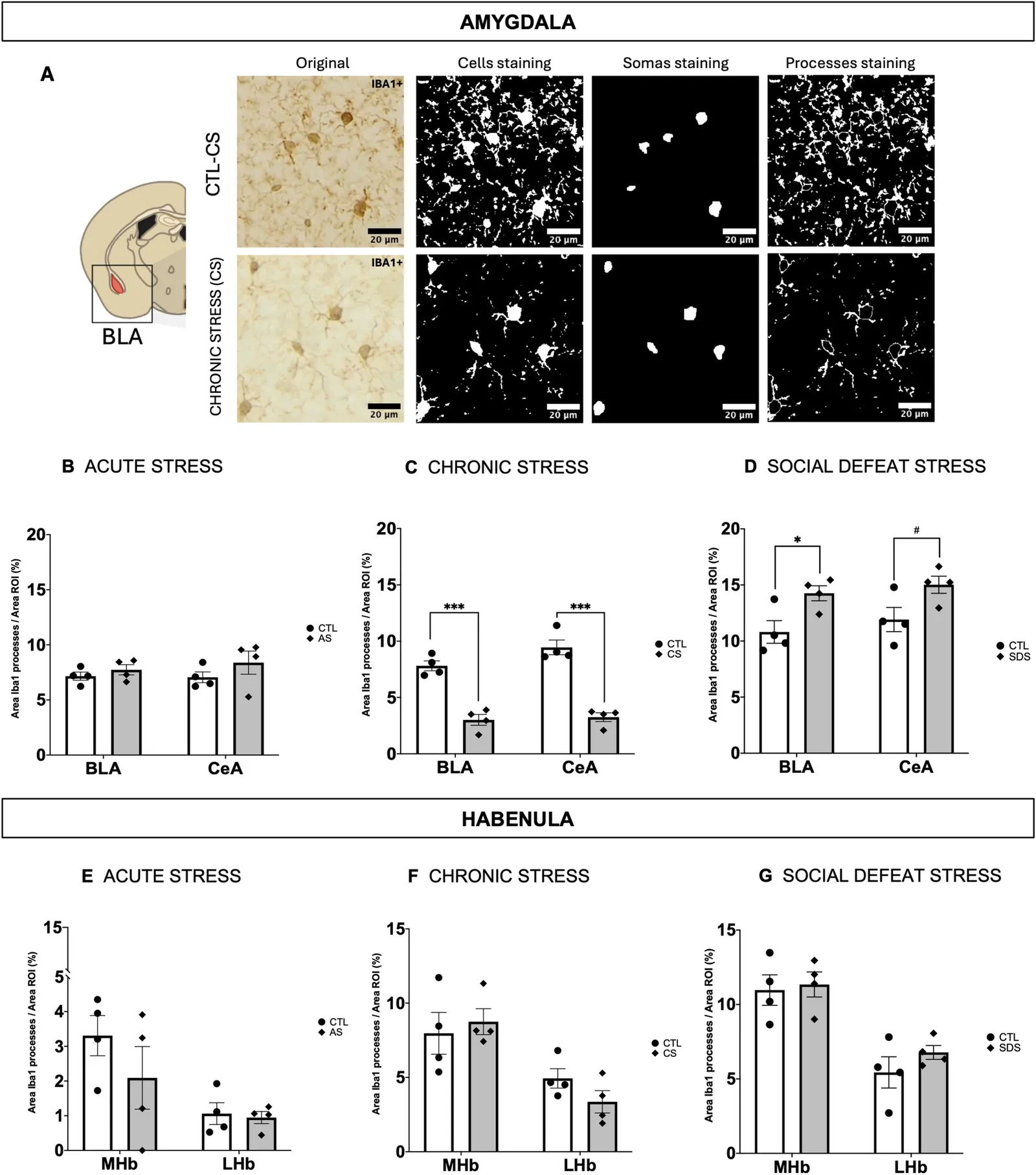
Microglial processes in Amygdala and Habenula subregions react differently to diverse stress protocols. (**A**) Representative images for microglial processes quantification in Basolateral Amygdala (BLA) under Chronic Stress paradigm (CS), as an example. Original IBA1 + images were binarized (black background) and somas staining (threshold 180) were substracted to cells staining (threshold 35, middle point in the range 25–45) to obtain processes staining. (**B**) Microglial processes in Amygdala subregions after Acute Stress (AS), measured as the mean percentage area of processes staining, quantified and averaged in the range of thresholds 25–45 and normalized by the area of the Region of Interest (ROI). Basolateral Amygdala (BLA): t(6.00) = -0.98; p = 0.37, N = 4; Central Amygdala (CeA): t(6.00) = -1.15; p = 0.29, N = 4; (**C**) Chronic stress (CS), BLA: t(6.00) = 7.34; p < 0.001, N = 4; CeA: t(6.00) = 8.14; p < 0.001, N = 4, (**D**) Social Defeat Stress (SDS), BLA: t(6.00) = -2.84; p = 0.030, N = 4; CeA: t(6.00) = -2.35; p = 0.057, N = 4; (**E**) Microglial processes in Habenula subregions after Acute Stress (AS), measured as the mean percentage area of processes staining, quantified and averaged in the range of thresholds 25–45 and normalized by the area of the Region of Interest (ROI). Medial Habenula (MHb): t(6.00) = 1.15; p = 0.30, N = 4, Lateral Habenula (LHb): t(6.00) = 0.32; p = 0.76, N = 4; (**F**) Chronic stress (CS), MHb: t(6.00) = -0.47; p = 0.65, N = 4; LHb: t(6.00) = 1.58; p = 0.17, N = 4; (**G**) Social Defeat Stress (SDS), MHb: t(6.00) = -0.29; p = 0.79, N = 4; LHb: t(6.00) = -1.17; p = 0.29, N = 4. Data are represented as mean ± SEM and analysed using Student’s t-test (two-tailed): * p < 0.05, ** p < 0.01, *** p < 0.001, ‘#’ for tendency within 0.05 < p < 0.1. N = 4 male mice per group per subregion in both Amygdala and Habenula. Each control group (CTL) within each stress paradigm constitutes a distinct control cohort specific to that experimental set

The results of the two-tailed Student’s/Welch’s t-tests for acute stress are presented in Tables 1 and 2 and Fig. 3. Among all the parameters analysed, only microglial density in the LHb showed a statistically significant difference. Acutely stressed animals exhibited lower microglial density in the LHb than control animals. In contrast, acute intense stress did not induce significant changes in any microglial morphometric parameter in the BLA, CeA, or MHb. No significant changes in the percentage of microglial processes were detected in any of the analysed subregions following acute stress.

**Table 1:** Results of Welch t-test (two-tailed) for microglial soma parameters of different subregions of the amygdala and habenula as a function of acute stress (PTSD-model)

	Control		Acute stress		*t*	*df*	*95% LL*	*95% UL*	*p*	*g*
	*M*	*SD*	*M*	*SD*						
*Amygdala*										
BLA Soma Area (µm^2^)	27.05	3.54	27.78	5.99	− 0.21	4.87	− 9.76	8.29	0.84	0.13
BLA Perimeter (µm)	21.68	1.75	22.28	2.99	− 0.32	4.84	− 5.05	3.96	0.77	0.19
BLA Circularity	0.73	0.02	0.72	0.03	0.39	4.93	− 0.04	0.06	0.71	0.24
BLA Feret diameter (µm)	8.21	0.60	8.28	0.95	− 0.14	5.06	− 1.51	1.36	0.90	0.09
BLA Aspect ratio	1.71	0.02	1.72	0.05	− 0.29	3.87	− 0.09	0.07	0.78	0.18
BLA Roundness	0.63	0.01	0.63	0.01	0.41	4.72	− 0.02	0.02	0.70	0.25
BLA Solidity	0.87	0.01	0.87	0.02	0.62	4.43	− 0.02	0.03	0.57	0.38
BLA Distance (µm)	42.94	9.39	42.97	11.82	− 0.01	5.71	− 18.74	18.67	0.99	0.00
BLA Density (soma/µm^2^)	2.421 × 10^–4^	9.256 × 10^–5^	2.715 × 10^–4^	1.168 × 10^–4^	− 0.39	5.70	− 25.40	1.552 × 10^–4^	0.71	0.24
CeA Soma Area (µm^2^)	28.18	4.32	28.22	5.51	− 0.01	5.68	− 8.73	8.65	0.99	0.01
CeA Perimeter (µm)	22.14	2.31	22.69	2.96	− 0.30	5.67	− 5.21	4.10	0.78	0.18
CeA Circularity	0.73	0.03	0.70	0.04	1.14	5.41	− 0.04	0.09	0.30	0.70
CeA Feret Diameter (µm)	8.35	0.80	8.47	0.95	− 0.19	5.84	− 1.64	1.41	0.86	0.12
CeA Aspect ratio	1.72	0.06	1.75	0.05	− 0.72	5.91	− 0.12	0.07	0.50	0.44
CeA Roundness	0.63	0.02	0.61	0.01	1.30	5.95	− 0.01	0.04	0.24	0.80
CeA Solidity	0.88	0.02	0.86	0.02	1.29	5.57	− 0.02	0.05	0.24	0.79
CeA Distance (µm)	39.91	10.59	40.81	11.05	− 0.12	5.99	− 19.64	17.83	0.91	0.07
CeA Density (soma/µm^2^)	2.685 × 10^–4^	1.203 × 10^–4^	2.693 × 10^–4^	1.146 × 10^–4^	− 0.01	5.99	− 24.43	2.026 × 10^–4^	0.99	0.01
*Habenula*										
MHb Soma Area (µm^2^)	34.03	6.59	34.87	4.28	− 0.21	5.15	− 10.85	9.19	0.84	0.13
MHb Perimeter (µm)	26.69	3.49	27.89	2.50	− 0.56	5.44	− 6.59	4.20	0.60	0.34
MHb Circularity	0.63	0.04	0.62	0.01	0.40	3.36	− 0.05	0.07	0.71	0.25
MHb Feret diameter (µm)	9.99	1.09	10.21	0.67	− 0.35	5.01	− 1.86	1.42	0.74	0.21
MHb Aspect ratio	2.09	0.11	2.09	0.02	0.07	3.19	− 0.18	0.18	0.95	0.04
MHb Roundness^a^	0.55	0.02	0.55	0.02	− 0.19	6.00	− 0.03	0.03	0.86	0.12
MHb Solidity	0.83	0.02	0.83	0.01	0.49	3.71	− 0.03	0.04	0.65	0.30
MHb Distance^a^ (µm)	28.9	5.48	29.46	5.38	− 0.14	6.00	− 9.95	8.84	0.89	0.09
MHb Density (soma/µm^2^)	4.797 × 10^–5^	1.679 × 10^–5^	4.008 × 10^–5^	1.562 × 10^–5^	0.69	5.97	− 25.20	3.598 × 10^–5^	0.52	0.42
LHb Soma Area (µm^2^)	30.01	6.84	24.39	4.94	1.33	5.46	− 4.96	16.19	0.24	0.82
LHb Perimeter (µm)	23.56	3.58	20.45	2.84	1.36	5.70	− 2.55	8.78	0.23	0.84
LHb Circularity	0.69	0.06	0.73	0.04	− 1.06	5.45	− 0.14	0.06	0.33	0.65
LHb Feret diameter (µm)	8.87	1.19	7.96	1.01	1.18	5.83	− 1.01	2.84	0.29	0.72
LHb Aspect Ratio	1.87	0.23	1.86	0.13	0.01	4.80	− 0.35	0.35	0.99	0.01
LHb Roundness	0.59	0.05	0.59	0.02	0.02	3.87	− 0.08	0.08	0.98	0.02
LHb Solidity	0.86	0.03	0.88	0.02	− 1.27	4.82	− 0.06	0.02	0.26	0.78
LHb Distance (µm)	42.74	13.41	95.71	52.89	− 1.94	3.38	− 134.47	28.54	0.14	1.19
LHb Density (soma/µm^2^)	3.205 × 10^–5^	1.430 × 10^–5^	9.114 × 10^–6^	4.558 × 10^–6^	**3.06**	**3.60**	**1.163 × 10** ^**–6**^	**4.471 × 10** ^**–5**^	**0.04**	**1.88**

^a^Student’s *t*-test; *n* = 4 animals per group. *M* = mean; *SD* = standard deviation; *t* = Welch’s *t* statistic; *df* = degree of freedom; *LL* = Lower limit; *UL* = Upper limit; *p* = significance level; *g* = effect size (Hedges’ *g*). *BLA*: Basolateral amygdala, *CeA*: Central amygdala, *MHb*: Medial habenula, *LHb*: Lateral habenula. Values in bold indicate statistically significant differences.Values in bold indicate statistically significant differences between stressed animals and their respective control groups

**Table 2:** Results of the Student’s t-test (two-tailed) for *microglial processes within different subregions of the amygdala and habenula as a function of* Acute Stress (PTSD-model)

	Control		Acute stress		*t*	*df*	*95% LL*	*95% UL*	*p*	*g*
	*M*	*SD*	*M*	*SD*						
*Amygdala*										
BLA Percentage of processes	7.16	0.74	7.74	0.93	− 0.98	6.00	− 2.04	0.88	.37	0.60
CeA Percentage of processes	7.06	0.98	8.39	2.10	− 1.15	6.00	− 4.17	1.50	.29	0.71
*Habenula*										
MHb Percentage of processes	3.31	1.16	2.03	1.90	1.15	6.00	− 1.44	3.99	.30	0.71
LHb Percentage of processes	1.06	0.63	0.95	0.35	0.32	6.00	− 0.77	0.99	.76	0.20

Levene’s test indicated homogeneity of variances across all groups and variables (all p > 0.05); *n* = 4 animals per group. *M* = mean; *SD* = standard deviation; *t* = Student’s *t* statistic; *df* = degree of freedom; *LL* = Lower limit; *UL* = Upper limit; *p* = significance level; *g* = effect size (Hedges’ *g*). BLA: Basolateral amygdala, CeA: Central amygdala, MHb: Medial habenula, LHb: Lateral habenula

### Chronic stress analysis

Tables 3 and 4 and Fig. 3 present the results of the two-tailed Student’s/Welch’s comparisons assessing the effects of chronic stress. Statistical analyses identified significant differences in eleven microglial morphometric parameters, indicating region-specific structural alterations associated with chronic stress exposure.

**Table 3:** Results of the Welch t-test (two-tailed) for microglial soma parameters of different subregions of the amygdala and habenula as a function of chronic stress

	Control		Chronic stress		*t*	*df*	*95% LL*	*95% UL*	*p*	*g*
	*M*	*SD*	*M*	*SD*						
*Amygdala*										
BLA Soma Area (µm^2^)	**26.97**	**2.82**	**33.83**	**3.90**	**− 2.86**	**5.46**	**− 12.89**	**− 0.84**	**0.03**	**1.75**
BLA Perimeter (µm)	**21.07**	**1.18**	**23.97**	**1.58**	**− 2.94**	**5.56**	**− 5.36**	**− 0.44**	**0.03**	**1.80**
BLA Circularity	0.76	4.33 × 10^–3^	0.74	0.01	2.41	3.50	**− **4.17 × 10^–3^	0.04	0.08	1.48
BLA Feret diameter (µm)	**8.02**	**0.36**	**9.07**	**0.59**	**− 3.05**	**5.01**	**-1.94**	**− 0.17**	**0.03**	**1.87**
BLA Aspect ratio	1.68	0.04	1.70	0.05	**− **0.63	5.79	**− **0.11	0.06	0.55	0.39
BLA Roundness	0.64	0.01	0.63	0.02	0.57	5.70	**− **0.02	0.03	0.59	0.35
BLA Solidity	0.89	0.00	0.89	0.01	1.47	5.46	**− **3.35 × 10^–3^	0.01	0.20	0.90
BLA Distance (µm)	34.19	2.06	35.44	4.46	**− **0.51	4.22	**− **7.93	5.43	0.64	0.31
BLA Density (soma/µm^2^)	3.48 × 10^–4^	7.07 × 10^–5^	3.21 × 10^–4^	7.48 × 10^–5^	0.53	5.98	**− **9.87 × 10^–5^	1.53 × 10^–4^	0.61	0.33
CeA Soma Area (µm^2^)	**29.33**	**2.90**	**35.43**	**3.49**	**− 2.69**	**5.81**	**− 11.69**	**− 0.51**	**0.04**	**1.65**
CeA Perimeter^a^ (µm)	**22.31**	**1.53**	**25.14**	**1.55**	**− 2.60**	**6.00**	**− 5.50**	**− 0.17**	**0.04**	**1.60**
CeA Circularity	0.74	0.02	0.72	0.02	1.69	5.99	**− **0.01	0.06	0.14	1.04
CeA Feret diameter (µm)	**8.40**	**0.52**	**9.48**	**0.60**	**− 2.74**	**5.88**	**− 2.06**	**− 0.11**	**0.03**	**1.68**
CeA Aspect ratio	1.69	0.07	1.77	0.09	**− **1.33	5.78	**− **0.22	0.07	0.23	0.82
CeA Roundness^a^	0.63	0.02	0.62	0.03	0.91	6.00	**− **0.03	0.06	0.40	0.56
CeA Solidity	0.88	0.01	0.87	0.01	1.15	5.99	**− **8.10 × 10^–3^	0.02	0.30	0.70
CeA Distance (µm)	33.33	2.14	38.48	12.86	**− **0.79	3.17	**− **25.29	14.99	0.48	0.49
CeA Density (soma/µm^2^)	3.76 × 10^–4^	6.56 × 10^–5^	2.97 × 10^–4^	8.69 × 10^–5^	1.44	5.58	**− **5.71 × 10^–5^	2.14 × 10^–4^	0.20	0.89
*Habenula*										
MHb Soma Area (µm^2^)	30.74	9.51	35.63	3.25	− 0.97	3.69	− 19.32	9.53	0.39	0.60
MHb Perimeter (µm)	22.67	4.51	27.08	1.98	− 1.79	4.12	− 11.17	2.35	0.15	1.10
MHb Circularity	**0.75**	**0.06**	**0.63**	**0.04**	**3.35**	**5.02**	**0.03**	**0.20**	**0.02**	**2.06**
MHb Feret diameter (µm)	8.30	1.50	10.08	0.70	− 2.15	4.25	− 4.03	0.47	0.09	1.32
MHb Aspectratio	**1.59**	**0.12**	**1.97**	**0.11**	− **4.77**	**5.94**	− **0.57**	− **0.18**	** < 0.001**	**2.93**
MHb Roundness	**0.66**	**0.04**	**0.57**	**0.02**	**4.00**	**4.58**	**0.03**	**0.16**	**0.01**	**2.46**
MHb Solidity	**0.88**	**0.02**	**0.83**	**0.02**	**3.83**	**5.45**	**0.02**	**0.09**	**0.01**	**2.35**
MHb Distance (µm)	52.71	24.64	32.65	5.01	1.60	3.25	− 18.28	58.40	0.20	0.98
MHb Density (soma/µm^2^)	**2.17 × 10** ^**–5**^	**1.12 × 10** ^**–5**^	**4.18 × 10** ^**–5**^	**8.97 × 10** ^**–6**^	− **2.80**	**5.72**	− **3.80 × 10**^**–5**^	− **2.35 × 10**^**–6**^	**0.03**	**1.72**
LHb Soma Area^a^ (µm^2^)	26.13	4.50	27.88	4.51	− 0.55	6.00	− 9.55	6.04	0.60	0.34
LHb Perimeter (µm)	21.08	2.02	22.09	2.72	− 0.60	5.54	− 5.24	3.22	0.57	0.37
LHb Circularity	0.74	0.05	0.73	0.05	0.23	5.98	− 0.08	0.10	0.83	0.14
LHb Feret diameter (µm)	8.11	0.75	8.37	0.90	− 0.45	5.82	− 1.70	1.18	0.67	0.28
LHb Aspect Ratio	1.79	0.26	1.75	0.11	0.24	4.08	− 0.36	0.43	0.82	0.15
LHb Roundness	0.62	0.08	0.62	0.03	− 0.04	3.90	− 0.12	0.11	0.97	0.03
LHb Solidity	0.89	4.71 × 10^–3^	0.88	0.02	0.88	3.22	− 0.03	0.05	0.44	0.54
LHb Distance (µm)	60.26	19.51	58.60	23.81	0.11	5.78	− 36.35	39.68	0.92	0.07
LHb Density (soma/µm^2^)	1.61 × 10^–5^	1.33 × 10^–5^	1.56 × 10^–5^	6.96 × 10^–6^	0.07	4.53	− 1.94 × 10^–5^	2.04 × 10^–5^	0.95	0.04

^a^Student’s *t*-test; *n* = 4 animals per group. *M* = mean; *SD* = standard deviation; *t* = Welch’s *t* statistic; *df* = degree of freedom; *LL* = Lower limit; *UL* = Upper limit; *p* = significance level; *g* = effect size (Hedges’ *g*). BLA: Basolateral amygdala, CeA: Central amygdala, MHb: Medial habenula, LHb: Lateral habenula. Values in bold indicate statistically significant differences.Values in bold indicate statistically significant differences between stressed animals and their respective control groups

**Table 4:** Results of the Student’s t-test (two-tailed) for microglial processes within different subregions of the amygdala and habenula as a function of Chronic Stress

	Control		Chronic Stress		*t*	*df*	*95% LL*	*95% UL*	*p*	*g*
	*M*	*SD*	*M*	*SD*						
*Amygdala*										
BLA percentage of processes	**7.81**	**0.89**	**3.01**	**0.97**	**7.34**	**6.00**	**3.20**	**6.41**	** < .001**	**4.51**
CeA percentage of processes	**9.45**	**1.31**	**3.25**	**0.78**	**8.14**	**6.00**	**4.34**	**8.07**	** < .001**	**5.01**
*Habenula*										
MHb percentage of processes	7.97	2.81	8.75	1.75	− 0.47	6.00	− 4.84	3.27	.65	0.29
LHb percentage of processes	4.94	1.31	3.36	1.51	1.58	6.00	− 0.87	4.02	.17	0.97

Levene’s test indicated homogeneity of variances across all groups and variables (all p > 0.05); *n* = 4 animals per group. *M* = mean; *SD* = standard deviation; *t* = Student’s *t* statistic; *df* = degree of freedom; *LL* = Lower limit; *UL* = Upper limit; *p* = significance level; *g* = effect size (Hedges’ *g*). *BLA* Basolateral amygdala, *CeA* Central amygdala, *MHb*: Medial habenula, *LHb* Lateral habenula. Values in bold indicate statistically significant differences between stressed animals and their respective control groups

Within the amygdala, both the BLA and the CeA exhibited increases in soma area, perimeter, and Feret diameter (Table 3), together with a significant reduction in the percentage of microglial processes (Table 4, Fig. 3). In the habenula, the MHb showed increases in aspect ratio and microglial density, along with decreases in circularity, roundness, and solidity (Table 3). However, no significant changes in the percentage of microglial processes were detected in the MHb (Table 4, Fig. 3). In the LHb, no significant changes were observed in either soma-related morphometric parameters or the percentage of microglial processes following chronic stress (Tables 3 and 4, Fig. 3).

### SDS stress analysis

Results from the two-tailed Student’s/Welch’s comparisons for SDS are presented in Tables 5 and 6 and Fig. 3. The analyses identified region-specific microglial morphological changes within the amygdala. In the amygdala, both the BLA and the CeA showed increases in soma area, perimeter, and Feret diameter. In addition, microglial density was increased in the BLA, whereas the CeA exhibited an increased aspect ratio. These changes were accompanied by reductions in circularity, roundness, and solidity in the CeA (Table 5).

**Table 5:** Results of the Welch t-test (two-tailed) for microglial soma parameters of different subregions of the amygdala and habenula as a function of SDS stress

	Control		SDS stress		*t*	*df*	*95% LL*	*95% UL*	*p*	*g*
	*M*	*SD*	*M*	*SD*						
*Amygdala*										
BLA Soma Area (µm^2^)	**37.27**	**2.10**	**40.7**	**0.41**	**− 3.21**	**3.23**	**− 6.70**	**− 0.16**	**0.04**	**1.97**
BLA Perimeter (µm)	**26.73**	**0.96**	**28.74**	**0.72**	**− 3.34**	**5.57**	**− 3.52**	**− 0.51**	**0.02**	**2.05**
BLA Circularity	0.67	0.01	0.64	0.02	2.34	5.32	**− **2.41 × 10^–3^	0.06	0.06	1.44
BLA Feret diameter (µm)	**9.95**	**0.30**	**10.53**	**0.17**	**− 3.34**	**4.80**	**− 1.02**	**− 0.13**	**0.02**	**2.05**
BLA Aspect ratio	1.82	0.01	1.84	0.04	**− **0.79	3.18	**− **0.08	0.05	0.48	0.49
BLA Roundness	0.60	< 0.01	0.60	0.01	0.41	3.81	**− **0.01	0.02	0.71	0.25
BLA Solidity	0.85	0.01	0.83	0.01	2.14	5.41	**− **3.16 × 10^–3^	0.04	0.08	1.32
BLA Distance (µm)	36.63	2.45	32.6	3.21	1.99	5.61	**− **1.01	9.05	0.10	1.22
BLA Density (soma/µm^2^)	**2.88 × 10**^**–4**^	**4.49 × 10**^**–5**^	**4.17 × 10**^**–4**^	**2.78 × 10**^**–5**^	**− 4.91**	**5.00**	**− 1.97 × 10**^**–4**^	**− 6.17 × 10**^**–5**^	** < 0.001**	**3.02**
CeA Soma Area (µm^2^)	**38.26**	**1.56**	**41.97**	**2.15**	**− 2.79**	**5.47**	**− 7.04**	**− 0.38**	**0.03**	**1.72**
CeA Perimeter (µm)	**26.80**	**0.29**	**29.22**	**1.08**	**− 4.32**	**3.42**	**− 4.09**	**− 0.75**	**0.02**	**2.65**
CeA Circularity	**0.68**	**0.01**	**0.63**	**0.01**	**4.62**	**5.98**	**0.02**	**0.07**	** < 0.001**	**2.84**
CeAFeret diameter (µm)	**9.94**	**0.15**	**10.65**	**0.39**	**− 3.41**	**3.87**	**− 1.29**	**− 0.12**	**0.03**	**2.10**
CeA Aspect ratio^a^	**1.76**	**0.02**	**1.82**	**0.02**	**− 3.38**	**6.00**	**− 0.10**	**− 0.02**	**0.01**	**2.08**
CeA Roundness	**0.62**	**0.01**	**0.60**	**0.01**	**4.05**	**5.98**	**9.24 × 10**^**–3**^	**0.04**	** < 0.001**	**2.49**
CeA Solidity	**0.86**	**0.01**	**0.83**	**0.01**	**5.10**	**5.57**	**0.01**	**0.04**	** < 0.001**	**3.13**
CeA Distance (µm)	**37.56**	**1.23**	**32.08**	**2.48**	**3.97**	**4.39**	**1.78**	**9.19**	**0.01**	**2.44**
CeA Density (soma/µm^2^)	3.08 × 10^–4^	2.26 × 10^–5^	3.40 × 10^–4^	4.48 × 10^–5^	**− **1.26	4.44	**− **9.87 × 10^–5^	3.53 × 10^–5^	0.27	0.78
*Habenula*										
MHb Soma Area (µm^2^)	42.51	4.65	42.85	4.42	**− **0.11	5.98	**− **8.20	7.52	0.92	0.07
MHb Perimeter (µm)	31.00	2.48	31.43	2.14	**− **0.26	5.87	**− **4.46	3.60	0.80	0.16
MHb Circularity	0.57	0.02	0.58	0.002	**− **081	3.08	**− **0.04	0.03	0.48	0.49
MHb Feret diameter (µm)	11.35	0.76	11.46	0.53	**− **0.24	5.37	**− **1.28	1.06	0.82	0.14
MHb Aspect ratio	2.07	0.08	2.06	0.02	0.19	3.27	**− **0.12	0.13	0.86	0.12
MHb Roundness	0.54	0.02	0.53	0.01	0.23	4.41	**− **0.03	0.03	0.83	0.14
MHb Solidity	0.80	0.01	0.79	0.01	0.40	3.73	**− **0.02	0.02	0.71	0.25
MHb Distance (µm)	33.13	4.63	29.58	3.19	1.26	5.33	**− **3.54	10.64	0.26	0.78
MHb Density (soma/µm^2^)	3.65 × 10^–5^	1.08 × 10^–5^	4.53 × 10^–5^	5.12 × 10^–6^	**− **1.46	4.29	**− **2.48 × 10^–5^	7.38 × 10^–6^	0.21	0.90
LHb Soma Area (µm^2^)	30.49	4.60	33.88	3.03	**− **1.23	5.19	**− **10.39	3.61	0.27	0.76
LHb Perimeter (µm)	23.52	2.30	25.86	1.68	**− **1.64	5.49	**− **5.91	1.23	0.16	1.01
LHb Circularity	0.70	0.03	0.65	0.03	2.17	5.99	**− **5.71 × 10^–3^	0.10	0.07	1.33
LHb Feret diameter (µm)	8.94	0.93	9.68	0.54	**− **1.37	4.85	**− **2.13	0.66	0.23	0.84
LHb Aspect Ratio	1.80	0.13	1.91	0.09	**− **1.32	5.35	**− **0.31	0.10	0.24	0.81
LHbRoundness	0.61	0.02	0.58	0.02	1.56	5.94	**− **0.01	0.06	0.17	0.96
LHb Solidity	0.86	0.01	0.84	0.02	1.73	5.65	**− **8.69 × 10^–3^	0.05	0.14	1.06
LHb Distance (µm)	53.93	11.22	43.75	2.82	1.76	3.38	**− **7.11	27.48	0.17	1.08
LHb Density (soma/µm^2^)	1.60 × 10^–5^	6.06 × 10^–6^	2.18 × 10^–5^	1.89 × 10^–6^	**− **1.81	3.58	**− **1.50 × 10^–5^	3.48 × 10^–6^	0.15	1.11

^a^ Student’s *t*-test; *n* = 4 animals per group. *M* = mean; *SD* = standard deviation; *t* = Welch’s *t* statistic; *df* = degree of freedom; *LL* = Lower limit; *UL* = Upper limit; *p* = significance level; *g* = effect size (Hedges’ *g*). BLA: Basolateral amygdala, CeA: Central amygdala, MHb: Medial habenula, LHb: Lateral habenulaValues in bold indicate statistically significant differences between stressed animals and their respective control groups

**Table 6:** Results of the Student’s t-test(two-tailed) for microglial processes within different subregions of the amygdala and habenula as a function of Social Defeat Stress

	Control		SDS Stress		*t*	*df*	*95% LL*	*95% UL*	*p*	*g*
	*M*	*SD*	*M*	*SD*						
*Amygdala*										
BLA percentage of processes	**10.80**	**2.02**	**14.30**	**1.35**	**− 2.84**	**6.00**	**− 6.42**	**-0.479**	**.030**	**1.75**
CeA percentage of processes	11.90	2.15	15.00	1.53	− 2.35	6.00	− 6.34	0.128	.057	1.44
*Habenula*										
MHb percentage of processes	11.00	2.05	11.03	1.68	− 0.29	6.00	− 3.62	2.86	.79	0.18
LHb percentage of processes	5.44	2.10	6.78	0.94	− 1.17	6.00	− 4.15	1.47	.29	0.72

Levene’s test indicated homogeneity of variances across all groups and variables (all p > 0.05); *n* = 4 animals per group. *M* = mean; *SD* = standard deviation; *t* = Student’s *t* statistic; *df* = degree of freedom; *LL* = Lower limit; *UL* = Upper limit; *p* = significance level; *g* = effect size (Hedges’ *g*). *BLA*: Basolateral amygdala, *CeA*: Central amygdala, *MHb*: Medial habenula, *LHb*: Lateral habenula. Values in bold indicate statistically significant differences between stressed animals and their respective control groups

Regarding microglial processes, SDS significantly increased the percentage of microglial processes in the BLA, whereas the CeA comparison did not reach statistical significance (p = 0.057) (Table 6, Fig. 3). In the habenula, SDS did not induce any significant effect on the microglial morphometric parameters analysed, including the percentage of microglial processes.

### K-means clustering

Section-level microglial morphometric profiles were classified into two exploratory clusters using k-means clustering based on soma area and percentage of process coverage. These two variables were selected as complementary soma- and process-related morphometric descriptors and were standardized as z-scores before clustering. Accordingly, cluster centroids and cluster summaries are reported in standardized units rather than raw physical units.

The clustering procedure was applied to individual tissue sections to retain local anatomical heterogeneity within each subregion. However, because sections were nested within animals, cluster membership was subsequently summarized at the animal level. For each animal, the proportion of sections assigned to each cluster was calculated as the number of sections assigned to that cluster divided by the total number of sections analysed for that animal and subregion. Group-level cluster distributions were then expressed as the mean ± SD of the four animal-level proportions. These animal-level summaries are reported in Table 7 for the amygdala, including the BLA and CeA, and the habenula, including the MHb and LHb. The corresponding animal-by-animal section counts, and cluster memberships are provided in Supplementary Table S3.

**Table 7:** Distribution of microglial morphometric clusters across brain regions, stress paradigms, and experimental groups after animal-level aggregation

Acute stress (AS)										
Region	Group	n animals	n sections (total)	Mean ± SD microglia per animal	Cluster 1 definition	Cluster 1 sections n (%)	Cluster 1 animal-level proportion mean ± SD (%)	Cluster 2 definition	Cluster 2 sections n (%)	Cluster 2 animal-level proportion mean ± SD (%)
BLA	Control	4	29	108.71 ± 43.61	Small soma/low processes	21 (72.4)	71.2 ± 12.7	Large soma/high processes	8 (27.6)	28.8 ± 12.7
BLA	Stressed	4	31	106.18 ± 47.60	Small soma/low processes	20 (64.5)	67.1 ± 23.8	Large soma/high processes	11 (35.5)	32.9 ± 23.8
CeA	Control	4	25	44.35 ± 24.16	Small soma/low processes	17 (68.0)	68.2 ± 37.4	Large soma/high processes	8 (32.0)	31.8 ± 37.4
CeA	Stressed	4	25	44.23 ± 21.25	Small soma/high processes	12 (48.0)	42.5 ± 43.5	Large soma/low processes	13 (52.0)	57.5 ± 43.5
MHb	Control	4	12	28.75 ± 9.99	Small soma/low processes	6 (50.0)	52.1 ± 44.3	Large soma/high processes	6 (50.0)	47.9 ± 44.3
MHb	Stressed	4	13	23.55 ± 6.10	Small soma/low processes	8 (61.5)	65.0 ± 47.3	Large soma/high processes	5 (38.5)	35.0 ± 47.3
LHb	Control	4	12	22.21 ± 16.45	Small soma/low processes	10 (83.3)	79.2 ± 25.0	Large soma/high processes	2 (16.7)	20.8 ± 25.0
LHb	Stressed	4	13	11.75 ± 9.4	Small soma/low processes	12 (92.3)	95.0 ± 10.0	Large soma/high processes	1 (7.7)	5.0 ± 10.0

Chronic stress (CS)										
Region	Group	n animals	n sections (total)	Mean ± SD microglia per animal	Cluster 1 definition	Cluster 1 sections n (%)	Cluster1 animal-level proportion mean ± SD (%)	Cluster 2 definition	Cluster 2 sections n (%)	Cluster 2 animal-level proportion mean ± SD (%)
BLA	Control	4	19	187.99 ± 71.42	Small soma/high processes	19 (100.0)	100.0 ± 0.0	Large soma/low processes	0 (0.0)	0.0 ± 0.0
BLA	Stressed	4	20	221.45 ± 101.43	Small soma/high processes	0 (0.0)	0.0 ± 0.0	Large soma/low processes	20 (100.0)	100.0 ± 0.0
CeA	Control	4	18	59.38 ± 15.45	Small soma/high processes	18 (100.0)	100.0 ± 0.0	Large soma/low processes	0 (0.0)	0.0 ± 0.0
CeA	Stressed	4	16	66.52 ± 31.90	Small soma/high processes	0 (0.0)	0.0 ± 0.0	Large soma/low processes	16 (100.0)	100.0 ± 0.0
MHb	Control	4	8	6.83 ± 3	Small soma/low processes	4 (50.0)	62.5 ± 47.9	Large soma/high processes	4 (50.0)	37.5 ± 47.9
MHb	Stressed	4	18	24.11 ± 4.8	Small soma/low processes	12 (66.7)	66.2 ± 32.0	Large soma/high processes	6 (33.3)	33.8 ± 32.0
LHb	Control	4	8	11.96 ± 9.4	Small soma/low processes	6 (75.0)	83.3 ± 33.3	Large soma/high processes	2 (25.0)	16.7 ± 33.3
LHb	Stressed	4	18	18.03 ± 10.24	Small soma/low processes	13 (72.2)	71.2 ± 21.7	Large soma/high processes	5 (27.8)	28.8 ± 21.7

Social defeat stress (SDS)										
Region	Group	n animals	n sections (total)	Mean ± SD microglia per animal	Cluster 1 definition	Cluster 1 sections n (%)	Cluster 1 animal-level proportion mean ± SD (%)	Cluster 2 definition	Cluster 2 sections n (%)	Cluster 2 animal-level proportion mean ± SD (%)
BLA	Control	4	28	139.50 ± 107.86	Small soma/low processes	17 (60.7)	60.2 ± 31.3	Large soma/high processes	11 ( 39.3)	39.8 ± 31.3
BLA	Stressed	4	31	165.63 ± 27.68	Small soma/low processes	5 (16.1)	15.8 ± 15.6	Large soma/high processes	26 (83.9)	84.2 ± 15.6
CeA	Control	4	27	44.00 ± 10.32	Small soma/low processes	16 (59.3)	58.3 ± 29.5	Large soma/high processes	11 (40.7)	41.7 ± 29.5
CeA	Stressed	4	26	42.90 ± 8.33	Small soma/low processes	5 (19.2)	19.8 ± 9.2	Large soma/high processes	21 (80.8)	80.2 ± 9.2
MHb	Control	4	18	20.95 ± 5.59	Small soma/low processes	13 (72.2)	68.3 ± 28.5	Large soma/high processes	5 (27.8)	31.7 ± 28.5
MHb	Stressed	4	19	23.15 ± 4.86	Small soma/low processes	9 (47.4)	48.8 ± 33.3	Large soma/high processes	10 (52.6)	51.2 ± 33.3
LHb	Control	4	18	15.57 ± 7.35	Small soma/low processes	8 (44.4)	40.0 ± 43.2	Large soma/high processes	10 (55.6)	60.0 ± 43.2
LHb	Stressed	4	18	21.78 ± 3.72	Small soma/low processes	3 (16.7)	16.2 ± 19.7	Large soma/high processes	15 (83.3)	83.8 ± 19.7

For each animal, the proportion of sections assigned to each cluster was first calculated. Group-level values are reported as mean ± SD of the four animal-level proportions; Cluster membership was summarized at the animal level to account for the nested structure of the data. For each animal, the animal-level Cluster 1 proportion was calculated as: number of sections assigned to Cluster 1 in that animal/total number of sections analysed in that animal. The same calculation was applied to Cluster 2. Group-level values are reported as the mean ± SD of the four animal-level proportions; For consistency across datasets, cluster labels were harmonized so that Cluster 1 corresponds to the lower soma-area centroid and Cluster 2 to the higher soma-area centroid

Because this clustering analysis was exploratory, cluster distributions were interpreted as descriptive summaries of morphometric profile membership rather than as confirmatory evidence of discrete microglial populations, functional phenotypes, or biological states.

Clustering performance was assessed using internal validation indices, including the Silhouette index, Calinski–Harabasz index, Davies–Bouldin index, and the proportion of variance explained by the clustering solution, estimated as BetweenSS/TotalSS. Across regions and stress paradigms, Silhouette values ranged from 0.36 to 0.64, indicating variable cluster separation. Cluster solutions with Silhouette values below 0.50 were considered to show weak separation and were therefore interpreted only as exploratorymorphometric partitions, rather than as robustly separated microglial profiles. Calinski–Harabasz indices ranged from 19 to 80, Davies–Bouldin indices from 0.84 to 1.20, and BetweenSS/TotalSS values from 0.36 to 0.52. Circularity and density, which were not used to generate the clustering solution, were examined only as additional descriptive morphometric comparisons between clusters. All clustering validation metrics, associated statistics, and cluster centroids are reported in Supplementary Table S1.

Animal-level bootstrap analyses showed acceptable to very high stability for most exploratory k-means partitions, particularly in BLA and CeA datasets, whereas SDS MHb showed the weakest stability. Some partitions showed variable or uneven cluster-wise stability and were therefore interpreted cautiously. Full ARI and Jaccard similarity metrics are reported in Supplementary Table S2. These analyses support the reproducibility of the exploratory morphometric partitions but do not constitute independent biological validation of discrete microglial states.

### Distribution of microglial clusters across brain regions and treatments

Cluster labels differed between brain regions due to region-specific distributions of soma area and process coverage. Accordingly, cluster numbering should not be compared directly across regions or paradigms. In the amygdala, clusters were generally defined as small soma/lower process coverage and large soma/higher process coverage. However, under chronic stress conditions, cluster labels in the amygdala corresponded to small soma/higher process coverage and large soma/lower process coverage, reflecting the distribution of sections within this specific condition. In the habenula, clusters were consistently defined as small soma/lower process coverage and large soma/higher process coverage. See Table 7 and Supplementary Table S3.

### Acute stress

Acute stress was associated with modest and region-dependent differences in exploratory microglial morphometric cluster distributions. In the BLA, the mean animal-level proportion of sections assigned to the large soma/high process coverage cluster increased slightly from 29% in control animals to 33% in acutely stressed animals, whereas the mean proportion assigned to the small soma/low process coverage cluster decreased from 71 to 67%.

In the CeA, cluster distributions showed a more marked descriptive redistribution. In control animals, the mean proportion of sections assigned to the small soma/low process coverage cluster was 68%, with 32% assigned to the large soma/high process coverage cluster. In acutely stressed animals, the mean proportion of sections was assigned to a large soma/low process coverage cluster (58%), whereas 43% were assigned to a small soma/high process coverage cluster.

In the MHb, the mean proportion of sections assigned to the large soma/high process coverage cluster decreased from 48% in control animals to 35% in acutely stressed animals, while the small soma/low process coverage cluster increased from 52 to 65%.

In the LHb, both groups were mainly represented by the small soma/low process coverage cluster. This mean proportion increased from 79% in controls to 95% in acutely stressed animals, whereas the large soma/high process coverage cluster decreased from 21 to 5%.

### Chronic stress

Under chronic stress, exploratory cluster distributions showed a clear descriptive redistribution in the amygdala. In the BLA, all sections from control animals were assigned to the small soma/high process coverage cluster (100%), whereas all sections from chronically stressed animals were assigned to the large soma/low process coverage cluster (100%).

In the CeA, the same descriptive pattern was observed. Sections from control animals were entirely assigned to the small soma/high process coverage cluster (100%), whereas sections from chronically stressed animals were entirely assigned to the large soma/low process coverage cluster (100%).

In the habenula, chronic stress was associated with more modest changes in cluster distribution. In the MHb, the mean animal-level proportion of sections assigned to the small soma/low process coverage cluster changed only slightly, from 63% in control animals to 66% in chronically stressed animals, while the large soma/high process coverage cluster decreased from 38 to 34%.

In the LHb, the mean animal-level proportion of sections assigned to the small soma/low process coverage cluster decreased from 83% in control animals to 71% in chronically stressed animals, whereas the large soma/high process coverage cluster increased from 17 to 29%.

### Social defeat stress (SDS)

Social defeat stress was associated with a marked descriptive redistribution of exploratory microglial morphometric clusters in the amygdala. In the BLA, the mean animal-level proportion of sections assigned to the large soma/high process coverage cluster increased from 40% in control animals to 84% in stressed animals, whereas the small soma/low process coverage cluster decreased from 60 to 16%.

A similar pattern was observed in the CeA, where the mean animal-level proportion of sections assigned to the large soma/high process coverage cluster increased from 42% in control animals to 80% following social defeat stress. Conversely, the small soma/low process coverage cluster decreased from 58 to 20%.

In the habenula, cluster distributions showed subregion-dependent descriptive changes. In the MHb, the mean animal-level proportion of sections assigned to the large soma/high process coverage cluster increased from 32% in control animals to 51% in stressed animals, while the small soma/low process coverage cluster decreased from 68 to 49%.

In the LHb, social defeat stress was associated with an increase in the mean animal-level proportion of sections assigned to the large soma/high process coverage cluster, from 60% in control animals to 84% in stressed animals. The small soma/low process coverage cluster decreased from 40 to 16%.

## Discussion

This study systematically examined stress-associated microglial morphometric changes in the amygdala and the habenula, two brain regions critically involved in emotional regulation. Although stress is a major environmental risk factor for mental health problems and microglia have been implicated in the etiopathogenesis of stress-related disorders, relatively few studies have directly examined how stress modifies microglial morphology within these structures (Hu et al. 2022; reviewed in Loonen 2023; O'Loughlin et al. 2017). Microglia show substantial morphological and functional diversity with responses that depend on the biological challenge and the regional tissue environment (Graeber and Streit 1990; Gomez-Nicola and Perry 2015; Ransohoff and Engelhardt 2012; Svahn et al. 2014; Wolf et al. 2017). In this context, the present study provides an exploratory, regionally resolved morphometric characterization of microglia in the basolateral and central amygdala (BLA and CeA) and in the medial and lateral habenula (MHb and LHb) after acute stress, chronic stress, and social defeat stress (SDS). Importantly, the findings are interpreted as morphometric observations, without assigning functional or biological state labels from morphology alone.

In the amygdala, microglial morphometric responses differed according to stressor type and subregion. Following acute stress, univariate analyses showed no significant changes in soma-related parameters or process coverage in either the BLA or the CeA. Exploratory clustering revealed only modest redistribution in the BLA and a descriptive shift in the CeA, but these findings were not accompanied by significant univariate amygdalar changes. Previous work has reported microglial changes in the CeA after acute restraint stress and related them to anxiety-like behaviours (Chen et al. 2024). In the present study, however, the data support a more restricted conclusion, indicating that acute stress produced limited amygdalar morphometric changes under the conditions analysed here. This limited response is compatible with the idea that short or intense stress exposure may induce early structural adjustments that are not consistently detected by individual morphometric endpoints. Previous studies have proposed that microglia are highly heterogeneous (Stratoulias et al. 2019; Healy et al. 2022; Dadwal and Heneka 2024) and that microglial morphology can change dynamically after stress, including modifications in process complexity or the emergence of intermediate morphologies under certain conditions (Fernández-Arjona et al. 2017; Ziebell et al. 2017; Woodburn et al. 2021; Dadwal and Heneka 2024). Nevertheless, in the present study these interpretations remain descriptive because no independent molecular or functional markers were included.

Chronic stress was associated with a clearer amygdalar pattern. In both the BLA and CeA, stress exposure increased soma size-related measures, including area,perimeter, and Feret diameter, and reduced process coverage. The exploratory cluster analysis paralleled these findings, showing a marked redistribution in both amygdalar subregions, with control sections mainly represented by small soma/high process coverage profiles and chronically stressed sections by large soma/low process coverage profiles. This convergence between univariate morphometry and cluster distribution suggests that chronic stress was associated with marked structural remodelling in the amygdala. Previous studies have similarly reported enlarged soma size and reduced microglial process length or branching after prolonged stress exposure (Tynan et al. 2010; Walker et al. 2013). Although these patterns are often discussed in relation to altered microglial physiology (Sanchez et al. 2022; Leyh et al. 2021; Madry et al. 2018; Savage et al. 2019; Lannes et al. 2017; Li et al. 2007; Lynch 2009), the present study restricts its conclusions to morphometric remodelling and does not infer specific microglial functional states.

SDS also produced prominent amygdalar changes. In the BLA, SDS increased soma area, perimeter, Feret diameter, microglial density, and process coverage. In the CeA, SDS increased soma area, perimeter, Feret diameter, and aspect ratio, and reduced circularity, roundness, solidity, and intercellular distance. These findings are consistent with previous descriptions of stress-associated structural microglial changes in limbic regions (Kreutzberg 1996; Wake et al. 2009; Vidal-Itriago et al. 2022). The CeA process comparison did not reach statistical significance and is therefore not interpreted as evidence of an effect. Exploratory clustering showed a descriptive increase in large soma/high process coverage profiles in both the BLA and CeA following SDS, supporting the view that SDS was associated with marked amygdalar morphometric remodelling. Previous work has shown that stress can be associated with increased microglial branching or process complexity in some contexts, including after SDS in relation to resilience-like outcomes (Fujikawa and Jinno 2022), and that species, strain, and stress modality can influence microglial morphology (Hinwood et al. 2012; 2013). The present findings are consistent with this broader variability, although they do not distinguish vulnerability- and resilience-related profiles.

Compared with the amygdala, the habenula showed a more restricted and subregion-dependent pattern. The LHb is a key regulator of negative motivational processing, emotional regulation, stress coping, and avoidance learning (Baker et al. 2022; Marks et al. 2022; Mondoloni et al. 2022), whereas the role of the MHb in stress-related processes remains less well characterized (Loonen 2023). The MHb also has distinct neuroanatomical, neurochemical, and immunological features and has received comparatively little attention in stress-related microglial studies (Antolín-Fontes et al. 2015). Analysing both habenular subdivisions separately was therefore important to avoid oversimplifying habenular microglial morphology.

After acute stress, the MHb showed no significant changes in individual morphometric parameters or process coverage. In the LHb, acute stress was associated with reduced microglial density, while other soma-related parameters and process coverage remained unchanged. Cluster distributions showed descriptive shifts in both habenular subregions, but these were not supported by broad univariate changes. Thus, the main acute stress-related habenular finding was the reduction in LHb microglial density. Under chronic stress, the MHb showed the clearest habenular changes, with reductions in circularity, roundness, and solidity, together with increases in aspect ratio and microglial density. However, process coverage did not change significantly in the MHb. In contrast, the LHb showed no significant soma-related changes or changes in process coverage following chronic stress. These results suggest that chronic stress affected MHb morphology more clearly than LHb morphology, whereas habenular cluster profiles remained comparatively constrained. Previous transcriptomic work has also shown that chronic restraint stress modifies gene expression profiles in the habenula, supporting the sensitivity of this region to prolonged stress exposure, although at a molecular rather than morphometric level (Yoo et al. 2022).

Following SDS, no significant soma-related or process-related changes were detected in either habenular subregion. Cluster distributions nevertheless showed descriptive shifts, especially in the LHb, where SDS was associated with a higher proportion of sections assigned to the large soma/high process coverage profile. Because these cluster findings were not accompanied by significant univariate habenular effects, they should be regarded as descriptive summaries rather than evidence of robust habenular remodelling**.** This distinction is important because the habenula, particularly the LHb, is increasingly implicated in stress-related and depressive-like circuitry (Hikosaka 2010; Balcita-Pedicino et al. 2011; Mohebi et al. 2019; Wang et al. 2023; Chengfeng et al. 2024), although the present study does not establish a direct mechanistic link between microglial morphology and circuit function.

More broadly, these findings support the view that stress-associated microglial morphology is shaped by anatomical context. The amygdala, owing to its established role in fear processing and regulation of negative affect, represents a key site where microglial structural remodelling may accompany stress-related changes in emotional processing (Wohleb et al. 2011; 2012; 2014). In parallel, habenular dysfunction has been implicated in psychiatric conditions and motivational disturbances (Hikosaka 2010), and previous studies have linked the acute stress paradigm used here to persistent anhedonia, increased avoidance behaviour, reduced appetite, and altered HPA-axis activity, resembling core features of PTSD (Nieto-Quero et al. 2023; Infantes-López et al. 2023). The MHb has also been implicated in anxiety-related processes (Wang et al. 2025). Nevertheless, although microglia are increasingly recognized as modulators of neural circuits and behaviour (Zhao et al. 2024), the present findings remain morphometric and descriptive, and do not establish causal relationships between microglial structure, neuronal activity, and behaviour.

The exploratory clustering analysis should be interpreted as a descriptive complement to the animal-level univariate analyses. Although cluster membership was aggregated at the animal level to account for the nested structure of the data, cluster labels were defined separately for each subregion and stress paradigm, and some solutions showed limited separation. Circularity and density were used only as additional morphometric descriptors to characterize the resulting clusters. Animal-level bootstrap analyses further indicated that several k-means partitions were reproducible under resampling, particularly in BLA and CeA datasets. However, bootstrap stability varied across regions and paradigms, with weaker or more variable stability in some habenular datasets, especially SDS MHb. Therefore, the bootstrap results support the internal reproducibility of several exploratory morphometric partitions, but they do not provide independent molecular, functional, or behavioural validation of discrete microglial states.

Several aspects should be considered when interpreting these findings. The sample size was modest, with four animals per group, which is common in detailed histological studies but may limit the detection of subtle effects. All experiments were conducted in male animals, and sex differences in microglial development, morphology, and stress responsivity may influence the generalizability of the results (Lynch 2022; Woodburn et al. 2021). The process coverage measure is an image-derived descriptor of Iba1-positive area and should not be interpreted as a full reconstruction of microglial arbor complexity. Finally, given the exploratory nature of the study and the number of related morphometric endpoints analysed, the results should be interpreted by emphasizing convergent patterns across related parameters rather than isolated statistically significant comparisons.

Despite these considerations, the present studyprovides a detailed regional map ofstress-associated microglial morphometric changes in two key emotional-regulation structures. The amygdala showed the most consistent remodelling, particularly after chronic stress and SDS, whereas habenular changes were more limited and subregion dependent. This region-specific pattern is in line with previous reports indicating that microglial responses to stress are heterogeneous across brain areas and experimental conditions (Furube et al. 2018; Jung et al. 2022; Tan et al. 2020; Vidal-Itriago et al. 2022). Importantly, the findings do not define discrete microglial states, but they identify anatomical and stressor-dependent morphometric patterns that can guide future mechanistic studies.

In conclusion, this exploratory study shows that stress is associated with region- and stressor-dependent microglial morphometric changes in the amygdala and habenula. Chronic stress and SDS produced the clearest amygdalar alterations, whereas habenular changes were more restricted and subregion specific. The cluster analysis provides an additional animal-level descriptive view of morphometric profile distributions, but it does not establish discrete biological microglial states. These findings provide a cautious anatomical framework for future studies integrating morphometry with molecular, functional, and behavioural approaches to determine the biological significance of stress-associated microglial structural changes and their possible contribution to stress-related vulnerability or resilience.

## Supplementary Information

Below is the link to the electronic supplementary material.

Supplementary file1 (PDF 12478 KB)

Supplementary file2 (PDF 26 KB)

Supplementary file3 (DOCX 61 KB)

## Data Availability

Data presented in this study are publicly available in the supplemental information of this publication, including numerical spreadsheets and macro code employed for soma quantification. Macro code for microglial processes quantification is deposited and made publicly available in GitHub (https://github.com/Jorvalgl/Thresholderer). The complete per-animal and per-section measurement datasets, including the individual measurements used in the analyses, are available from the corresponding author upon reasonable request. Access will be provided through a maintained data link.

## References

[CR1] Adusah AK, Brooks GP (2011) Type I error inflation of the separate-variances Welch t test with very small sample sizes when assumptions are met. J Mod Appl Stat Methods 10(1):362–372. 10.22237/jmasm/1304224320

[CR2] Antolin-Fontes B, Ables JL, Görlich A, Ibañez-Tallon I (2015) The habenulo-interpeduncular pathway in nicotine aversion and withdrawal. Neuropharmacology 96(Part B):213–222. 10.1016/j.neuropharm.2014.11.01925476971 PMC4452453

[CR3] Asim M, Wang H, Waris A, Zhang X, Liu J (2024) Basolateral amygdala parvalbumin and cholecystokinin-expressingGABAergic neurons modulate depressive and anxiety-like behaviors. Transl Psychiatry 14:418. 10.1038/s41398-024-03135-z39368965 PMC11455908

[CR4] Baker PM, Mathis V, Lecourtier L, Simmons SC, Nugent FS, Hill S, Mizumori SJY (2022) Lateral habenula beyond avoidance: roles in stress, memory, and decision-making with implications for psychiatric disorders. Front Syst Neurosci 16:826475. 10.3389/fnsys.2022.82647535308564 PMC8930415

[CR5] Balcita-Pedicino JJ, Omelchenko N, Bell R, Sesack SR (2011) The inhibitory influence of the lateral habenula on midbrain dopamine cells: ultrastructural evidence for indirect mediation via the rostromedial mesopontine tegmental nucleus. J Comp Neurol 519(6):1143–1164. 10.1002/cne.2256121344406 PMC4054696

[CR6] Charlson F, van Ommeren M, Flaxman A, Cornett J, Whiteford H, Saxena S (2019) New WHO prevalence estimates of mental disorders in conflict settings: a systematic review and meta-analysis. Lancet 394:240–248. 10.1016/S0140-6736(19)30934-131200992 PMC6657025

[CR7] Chattarji S, Tomar A, Suvrathan A, Ghosh S, Rahman MM (2015) Neighborhood matters: divergent patterns of stress-induced plasticity across the brain. Nat Neurosci 18(10):1364–1375. 10.1038/nn.4115 26404711

[CR8] Chen D, Lou Q, Song XJ, Kang F, Liu A, Zheng C, Li Y, Wang D, Qun S, Zhang Z, Cao P, Jin Y (2024) Microglia govern the extinction of acute stress-induced anxiety-like behaviors in male mice. Nat Commun 15(1):449. 10.1038/s41467-024-44704-638200023 PMC10781988

[CR9] Chengfeng C, Mingqia W, Tong Y, Wanting F, Yingyi X, Yuping N, Bin Z (2024) Habenular functional connections are associated with depression state and modulated by ketamine. J Affect Disord 345:177–185. 10.1016/j.jad.2023.10.136 37879411

[CR10] Cohen J (1988) Statistical power analysis for the behavioral sciences, 2nd ed. Lawrence Erlbaum Associates

[CR11] Companion MA, Gonzalez DA, Robinson SL, Herman MA, Thiele TE (2022) Lateral habenula-projecting central amygdala circuits expressing GABA and NPY Y1 receptor modulate binge-like ethanol intake in mice. Addict Neurosci 3:100019.10.1016/j.addicn.2022.10001936059430 PMC9435303

[CR12] Dadwal S, Heneka MT (2024) Microglia heterogeneity in health and disease. FEBS Open Bio 14(2):217–229. 10.1002/2211-5463.1373537945346 PMC10839410

[CR13] Davis BM, Salinas-Navarro M, Cordeiro MF, Moons L, De Groef L (2017) Characterizing microglia activation: a spatial statistics approach to maximize information extraction. Sci Rep 7:Article 1576. 10.1038/s41598-017-01747-828484229 PMC5431479

[CR14] Duvarci S, Paré D (2014) Amygdala microcircuits controlling learned fear. Neuron 82(5):966–980. 10.1016/j.neuron.2014.04.04224908482 PMC4103014

[CR15] Fernández-Arjona MDM, Grondona JM, Granados-Durán P, Fernández-Llebrez P, López-Ávalos MD (2017) Microglia morphological categorization in a rat model of neuroinflammation by hierarchical cluster and principal components analysis. Front Cell Neurosci 11:235. 10.3389/fncel.2017.0023528848398 PMC5550745

[CR16] Fowler CD, Tuesta L, Kenny PJ (2013) Role of α5 nicotinic acetylcholine receptors in the effects of acute and chronic nicotine treatment on brain reward function in mice. Psychopharmacology Berl 229(3):503–513. 10.1007/s00213-013-3235-1 PMC3930613 23958943

[CR17] Fujikawa R, Jinno S (2022) Identification of hyper-ramified microglia in the CA1 region of the mouse hippocampus potentially associated with stress resilience. Eur J Neurosci 56(8):5137–5153. 10.1111/ejn.15812 36017697

[CR18] Furube E, Kawai S, Inagaki H, Takagi S, Miyata S (2018) Brain region-dependent heterogeneity and dose-dependent difference in transient microglia population increase during lipopolysaccharide-induced inflammation. Sci Rep 8:Article 2203. 10.1038/s41598-018-20643-329396567 PMC5797160

[CR19] Gebresenbet EA, Zegeye S, Biratu TD (2005) Prevalence and associated factors of depression and posttraumatic stress disorder among trauma patients: multi-centered cross-sectional study. Front Psychiatry 16:1447232. 10.3389/fpsyt.2025.1447232 PMC11925855 40123599

[CR20] Golden SA, Covington HE III, Berton O, Russo SJ (2011) A standardized protocol for repeated social defeat stress in mice. Nat Protoc 6(8):1183–1191. 10.1038/nprot.2011.36121799487 PMC3220278

[CR21] Gomez-Nicola D, Perry VH (2015) Microglial dynamics and role in the healthy and diseased brain: a paradigm of functional plasticity. Neuroscientist 21(2):169–184. 10.1177/107385841453051224722525 PMC4412879

[CR22] Graeber MB, Streit WJ (1990) Perivascular microglia defined. Trends Neurosci 13(9):366. 10.1016/0166-2236(90)90020-B 1699325

[CR23] Hartmann SM, Heider J, Wüst R, Fallgatter AJ, Volkmer H (2024) Microglia-neuron interactions in schizophrenia. Front Cell Neurosci 18:1345349. 10.3389/fncel.2024.1345349 PMC10950997 38510107

[CR24] Healy LM, Zia S, Plemel JR (2022) Towards a definition of microglia heterogeneity. Commun Biol 5:1114. 10.1038/s42003-022-04081-636266565 PMC9585025

[CR25] Hedges LV, Olkin I (1985) Statistical methods for meta-analysis. Academic Press. 10.1016/C2009-0-03396-0

[CR26] Hikosaka O (2010) The habenula: from stress evasion to value-based decision-making. Nat Rev Neurosci 11(7):503–513. 10.1038/nrn286620559337 PMC3447364

[CR27] Hinwood M, Morandini J, Day TA, Walker FR (2012) Evidence that microglia mediate the neurobiological effects of chronic psychological stress on the medial prefrontal cortex. Cereb Cortex 22(6):1442–1454. 10.1093/cercor/bhr229 21878486

[CR28] Hinwood M, Tynan RJ, Charnley JL, Beynon SB, Day TA, Walker FR (2013) Chronic stress induced remodeling of the prefrontal cortex: structural re-organization of microglia and the inhibitory effect of minocycline. Cereb Cortex 23(8):1784–1797. 10.1093/cercor/bhs151 22710611

[CR29] Hsu Y-WA, Wang SD, Wang S, Morton G, Zariwala HA, de la Iglesia HO, Turner EE (2014) Role of the dorsal medial habenula in the regulation of voluntary activity, motor function, hedonic state, and primary reinforcement. J Neurosci 34(34):11366–11384. 10.1523/JNEUROSCI.1861-14.201425143617 PMC4138345

[CR30] Hu H, Cui Y, Yang Y (2020) Circuits and functions of the lateral habenula in health and in disease. Nat Rev Neurosci 21(5):277–295. 10.1038/s41583-020-0292-4 32269316

[CR31] Hu P, Lu Y, Pan B-X, Zhang W-H (2022) New insights into the pivotal role of the amygdala in inflammation-related depression and anxiety disorder. Int J Mol Sci 23(19):11076. 10.3390/ijms23191107636232376 PMC9570160

[CR32] Infantes-López MI, Nieto-Quero A, Chaves-Peña P, Zambrana-Infantes E, Cifuentes M, Márquez J, Pedraza C, Pérez-Martín M (2023) New insights into hypothalamic neurogenesis disruption after acute and intense stress: implications for microglia and inflammation. Front Neurosci 17:1190418. 10.3389/fnins.2023.119041837425000 PMC10327603

[CR33] Jacinto LR, Mata R, Novais A, Marques F, Sousa N (2017) The habenula as a critical node in chronic stress-related anxiety. Exp Neurol 289:46–54. 10.1016/j.expneurol.2016.12.003 27940019

[CR34] JASP Team (2023) JASP (Version 0.17.2) [Computer software]. https://jasp-stats.org/

[CR35] Jung H, Lee H, Kim D, Cheong E, Hyun YM, Yu JW, Um JW (2022) Differential regional vulnerability of the brain to mild neuroinflammation induced by systemic LPS treatment in mice. J Inflamm Res 15:3053–3063. 10.2147/JIR.S36200635645573 PMC9140139

[CR36] Kobayashi Y, Sano Y, Vannoni E, Goto H, Suzuki H, Oba A, Kawasaki H, Kanba S, Lipp H-P, Murphy NP, Wolfer DP, Itohara S (2013) Genetic dissection of medial habenula-interpeduncular nucleus pathway function in mice. Front Behav Neurosci 7:17. 10.3389/fnbeh.2013.0001723487260 PMC3594921

[CR37] Kreisel T, Frank MG, Licht T, Reshef O, Ben-Menachem-Zidon O, Baratta MV, Maier SF, Yirmiya R (2014) Dynamic microglial alterations underlie stress-induced depressive-like behavior and suppressed neurogenesis. Mol Psychiatry 19(6):699–709. 10.1038/mp.2013.155 24342992

[CR38] Kreutzberg GW (1996) Microglia: a sensor for pathological events in the CNS. Trends Neurosci 19(8):312–318. 10.1016/0166-2236(96)10049-7 8843599

[CR39] Kupferberg A, Hasler G (2023) The social cost of depression: investigating the impact of impaired social emotion regulation, social cognition, and interpersonal behavior on social functioning. J Affect Disord Rep 14:100631. 10.1016/j.jadr.2023.100631

[CR40] Lannes N, Eppler E, Etemad S, Yotovski P, Filgueira L (2017) Microglia at center stage: acomprehensive review about the versatile and unique residential macrophages of the central nervous system. Oncotarget 8:114393–114413. 10.18632/oncotarget.2310629371994 PMC5768411

[CR41] Lecourtier L, Neijt H, Kelly P (2004) Habenula lesions cause impaired cognitive performance in rats: implications for schizophrenia. Eur J Neurosci 19(9):2551–2560. 10.1111/j.0953-816X.2004.03356.x 15128408

[CR42] Lee Y, Goto Y (2011) Neurodevelopmental disruption of cortico-striatal function caused by degeneration of habenula neurons. PLoS ONE 6(4):e19450. 10.1371/journal.pone.001945021559387 PMC3084869

[CR43] Lee EH, Huang SL (1988) Role of lateral habenula in the regulation of exploratory behavior and its relationship to stress in rats. Behav Brain Res 30(3):265–271. 10.1016/0166-4328(88)90169-6 3178997

[CR44] Leyh J, Paeschke S, Mages B, Michalski D, Nowicki M, Bechmann I, Winter K (2021) Classification of microglial morphological phenotypes using machine learning. Front Cell Neurosci 15:701673. 10.3389/fncel.2021.70167334267628 PMC8276040

[CR45] Li Y, Chu N, Hu A, Gran B, Rostami A, Zhang G-X (2007) Increased IL-23p19 expression in multiple sclerosis lesions and its induction in microglia. Brain 130:490–501. 10.1093/brain/awl273 17003070

[CR46] Loonen AJM (2023) Role of neuroglia in the habenular connection hub of the dorsal diencephalic conduction system. Neuroglia 4(1):34–51. 10.3390/neuroglia4010004

[CR47] Lynch MA (2009) The multifaceted profile of activated microglia. Mol Neurobiol 40:139–156. 10.1007/s12035-009-8077-9 19629762

[CR48] Lynch MA (2022) Exploring sex-related differences in microglia may be a game-changer in precision medicine. Front Aging Neurosci 14:868448. 10.3389/fnagi.2022.86844835431903 PMC9009390

[CR49] Madry C, Arancibia-Cárcamo IL, Kyrargyri V, Chan VTT, Hamilton NB, Attwell D (2018) Effects of the ecto-ATPase apyrase on microglial ramification and surveillance reflect cell depolarization, not ATP depletion. Proc Natl Acad Sci U S A 115(7):E1608–E1617. 10.1073/pnas.171535411529382767 PMC5816168

[CR50] Maren S, Quirk GJ (2004) Neuronal signalling of fear memory. Nat Rev Neurosci 5(11):844–852. 10.1038/nrn1535 15496862

[CR51] Marks RB, Wee JY, Jacobson SV, Hashimoto K, O’Connell KL, Golden SA, Baker PM, Law KC (2022) The role of the lateral habenula in suicide: a call for further exploration. Front Behav Neurosci 16:812952. 10.3389/fnbeh.2022.81295235359586 PMC8964288

[CR52] McEwen BS (2007) Physiology and neurobiology of stress and adaptation: central role of the brain. Physiol Rev 87(3):873–904. 10.1152/physrev.00041.2006 17615391

[CR53] McEwen BS, Nasca C, Gray JD (2016) Stress effects on neuronal structure: hippocampus, amygdala, and prefrontal cortex. Neuropsychopharmacology 41(1):3–23. 10.1038/npp.2015.17126076834 PMC4677120

[CR54] Mohebi A, Pettibone JR, Hamid AA, Wong J-MT, Vinson LT, Patriarchi T, Tian L, Kennedy RT, Berke JD (2019) Dissociable dopamine dynamics for learning and motivation. Nature 570(7759):65–70. 10.1038/s41586-019-1235-y31118513 PMC6555489

[CR55] Mondoloni S, Mameli M, Congiu M (2022) Reward and aversion encoding in the lateral habenula for innate and learned behaviours. Transl Psychiatry 12:3. 10.1038/s41398-021-01774-035013094 PMC8748902

[CR56] Moreno-Fernández RD, Rosell-Valle C, Bacq A, Zanoletti O, Cifuentes M, Pérez-Martín M, Gavito AL, García-Fernández MI, Estivill-Torrús G, Rodríguez de Fonseca F, Santín LJ, Sandi C, Pedraza C (2020) LPA1 receptor and chronic stress: effects on behaviour and the genes involved in the hippocampal excitatory/inhibitory balance. Neuropharmacology 164:107896. 10.1016/j.neuropharm.2019.107896 31811875

[CR57] Murphy CA, DiCamillo AM, Haun F, Murray M (1996) Lesion of the habenular efferent pathway produces anxiety and locomotor hyperactivity in rats: a comparison of the effects of neonatal and adult lesions. Behav Brain Res 81(1–2):43–52. 10.1016/S0166-4328(96)00041-1 8950000

[CR58] Nieto-Quero A, Chaves-Peña P, Santín LJ, Pérez-Martín M, Pedraza C (2021) Do changes in microglial status underlie neurogenesis impairments and depressive-like behaviours induced by psychological stress? A systematic review in animal models. Neurobiol Stress 15:100356.10.1016/j.ynstr.2021.10035634355047 PMC8319800

[CR59] Nieto-Quero A, Infantes-López MI, Zambrana-Infantes E, Chaves-Peña P, Gavito AL, Munoz-Martin J, Tabbai S, Márquez J, Rodríguez de Fonseca F, García-Fernández MI, Santín LJ, Pedraza C, Pérez-Martín M (2023) Unveiling the secrets of the stressed hippocampus: exploring proteomic changes and neurobiology of posttraumatic stress disorder. Cells 12(18):2290. 10.3390/cells1218229037759512 PMC10527244

[CR60] O’Connor JL, Nissen JC (2023) The pathological activation of microglia is modulated by sexually dimorphic pathways. Int J Mol Sci 24(5):4739. 10.3390/ijms2405473936902168 PMC10003784

[CR61] O’Loughlin E, Pakan JMP, Yilmazer-Hanke D, McDermott KW (2017) Acute in utero exposure to lipopolysaccharide induces inflammation in the pre- and postnatal brain and alters the glial cytoarchitecture in the developing amygdala. J Neuroinflammation 14:212. 10.1186/s12974-017-0981-829096641 PMC5667487

[CR62] Ohgidani M, Kato TA, Sagata N, Hayakawa K, Shimokawa N, Sato-Kasai M, Kanba S (2016) TNF-α from hippocampal microglia induces working memory deficits by acute stress in mice. Brain Behav Immun 55:17–24. 10.1016/j.bbi.2015.08.022 26551431

[CR63] Pallarés-Moratalla C, Bergers G (2024) The ins and outs of microglial cells in brain health and disease. Front Immunol 15:1305087. 10.3389/fimmu.2024.130508738665919 PMC11043497

[CR64] Paxinos G, Franklin KBJ (2004) The mouse brain in stereotaxic coordinates, 2nd ed. Academic Press

[CR65] Piper JA, Musumeci G, Castorina A (2024) The neuroanatomy of the habenular complex and its role in the regulation of affective behaviors. J Funct Morphol Kinesiol 9(1):14. 10.3390/jfmk901001438249091 PMC10801627

[CR66] Proulx CD, Hikosaka O, Malinow R (2014) Reward processing by the lateral habenula in normal and depressive behaviors. Nat Neurosci 17(9):1146–1152. 10.1038/nn.377925157511 PMC4305435

[CR67] Ransohoff RM, Engelhardt B (2012) The anatomical and cellular basis of immune surveillance in the central nervous system. Nat Rev Immunol 12(9):623–635. 10.1038/nri3265 22903150

[CR68] Sanchez K, Darling JS, Kakkar R, Wu SL, Zentay A, Lowry CA, Fonken LK (2022) *Mycobacterium vaccae* immunization in rats ameliorates features of age-associated microglia activation in the amygdala and hippocampus. Sci Rep 12:2165. 10.1038/s41598-022-05275-y35140249 PMC8828872

[CR69] Savage JC, Carrier M, Tremblay M-È (2019) Morphology of microglia across contexts of health and disease. In: Methods in Molecular Biology (Vol. 2034, pp. 13–26). 10.1007/978-1-4939-9658-2_2 31392674

[CR70] Schneider CA, Rasband WS, Eliceiri KW (2012) NIH image to ImageJ: 25 years of image analysis. Nat Methods 9(7):671–675. 10.1038/nmeth.208922930834 PMC5554542

[CR71] Schneiderman N, Ironson G, Siegel SD (2005) Stress and health: psychological, behavioral, and biological determinants. Annu Rev Clin Psychol 1:607–628. 10.1146/annurev.clinpsy.1.102803.14414117716101 PMC2568977

[CR72] Steinberg E, Gore F, Heifets B, Taylor M, Norville Z, Beier K, Földy C, Lerner T, Luo L, Deisseroth K, Malenka R (2020) Amygdala-midbrain connections modulate appetitive and aversive learning. Neuron 106(1):102–115. 10.1016/j.neuron.2020.03.016 32294466

[CR73] Stratoulias V, Venero JL, Tremblay MÈ, Joseph B (2019) Microglial subtypes: diversity within the microglial community. EMBO J 38(17):e101997. 10.15252/embj.201910199731373067 PMC6717890

[CR74] Svahn AJ, Becker TS, Graeber MB (2014) Emergent properties of microglia. Brain Pathol 24(6):665–670. 10.1111/bpa.1219525345896 PMC8029137

[CR75] Tan YL, Yuan Y, Tian L (2020) Microglial regional heterogeneity and its role in the brain. Mol Psychiatry 25:351–367. 10.1038/s41380-019-0609-831772305 PMC6974435

[CR76] Tay TL, Béchade C, D’Andrea I, St-Pierre MK, Henry MS, Roumier A, Tremblay MÈ (2018) Microglia gone rogue: impacts on psychiatric disorders across the lifespan. Front Mol Neurosci 10:421. 10.3389/fnmol.2017.0042129354029 PMC5758507

[CR77] Thornton EW, Murray M, Connors-Eckenrode T, Haun F (1994) Dissociation of behavioral changes in rats resulting from lesions of the habenula versus fasciculus retroflexus and their possible anatomical substrates. Behav Neurosci 108(6):1150–1162. 10.1037/0735-7044.108.6.1150 7893407

[CR78] Tye KM, Prakash R, Kim S-Y, Fenno LE, Grosenick L, Zarabi H, Thompson KR, Gradinaru V, Ramakrishnan C, Deisseroth K (2011) Amygdala circuitry mediating reversible and bidirectional control of anxiety. Nature 471(7338):358–362. 10.1038/nature0982021389985 PMC3154022

[CR79] Tynan RJ, Naicker S, Hinwood M, Nalivaiko E, Buller KM, Pow DV, Day TA, Walker FR (2010) Chronic stress alters the density and morphology of microglia in a subset of stress-responsive brain regions. Brain Behav Immun 24(7):1058–1068. 10.1016/j.bbi.2010.02.001 20153418

[CR80] Ulrich-Lai YM, Herman JP (2009) Neural regulation of endocrine and autonomic stress responses. Nat Rev Neurosci 10(6):397–409. 10.1038/nrn264719469025 PMC4240627

[CR81] Veinante P, Yalcin I, Barrot M (2013) The amygdala between sensation and affect: a role in pain. J Mol Psychiatry 1(1):9. 10.1186/2049-9256-1-925408902 PMC4223879

[CR82] Vidal-Itriago A, Radford RAW, Aramideh JA, Maurel C, Scherer NM, Don EK, Lee A, Chung RS, Graeber MB, Morsch M (2022) Microglia morphophysiological diversity and its implications for the CNS. Front Immunol 13:997786. 10.3389/fimmu.2022.99778636341385 PMC9627549

[CR83] Wake H, Moorhouse AJ, Jinno S, Kohsaka S, Nabekura J (2009) Resting microglia directly monitor the functional state of synapses in vivo and determine the fate of ischemic terminals. J Neurosci 29(13):3974–3980. 10.1523/JNEUROSCI.4363-08.200919339593 PMC6665392

[CR84] Walker FR, Nilsson M, Jones K (2013) Acute and chronic stress-induced disturbances of microglial plasticity, phenotype and function. Curr Drug Targets 14(11):1262–1276. 10.2174/1389450111314999020824020974 PMC3788324

[CR85] Wang XY, Xu X, Chen R, Jia WB, Xu PF, Liu XQ, Zhang Y, Liu XF, Zhang Y (2023) The thalamic reticular nucleus-lateral habenula circuit regulates depressive-like behaviors in chronic stress and chronic pain. Cell Rep 42(10):113170. 10.1016/j.celrep.2023.113170 37738124

[CR86] Wang Y, Lv XY, Qiao JY et al (2025) A medial habenula neural circuit controlling anxiety-like behaviors in response to acute stress. Mol Psychiatry 30:5245–5254. 10.1038/s41380-025-03111-z 40659840

[CR87] White AG, Elias E, Orozco A, Robinson SA, Manners MT (2024) Chronic stress-induced neuroinflammation: relevance of rodent models to human disease. Int J Mol Sci 25(10):5085. 10.3390/ijms2510508538791125 PMC11121038

[CR88] Wohleb ES (2016) Neuron–microglia interactions in mental health disorders: “for better, and for worse.” Front Immunol 7:544. 10.3389/fimmu.2016.0054427965671 PMC5126117

[CR89] Wohleb ES, Hanke ML, Corona AW, Powell ND, Stiner LM, Bailey MT, Nelson RJ, Godbout JP, Sheridan JF (2011) β-Adrenergic receptor antagonism prevents anxiety-like behavior and microglial reactivity induced by repeated social defeat. J Neurosci 31(17):6277–6288. 10.1523/JNEUROSCI.0450-11.201121525267 PMC3160240

[CR90] Wohleb ES, Fenn AM, Pacenta AM, Powell ND, Sheridan JF, Godbout JP (2012) Peripheral innate immune challenge exaggerated microglia activation, increased the number of inflammatory CNS macrophages, and prolonged social withdrawal in socially defeated mice. Psychoneuroendocrinology 37(9):1491–1505. 10.1016/j.psyneuen.2012.02.00322386198 PMC3368999

[CR91] Wohleb ES, McKim DB, Shea DT, Powell ND, Tarr AJ, Sheridan JF, Godbout JP (2014) Re-establishment of anxiety in stress-sensitized mice is caused by monocyte trafficking from the spleen to the brain. Biol Psychiatry 75(12):970–981. 10.1016/j.biopsych.2013.11.02924439304 PMC4084643

[CR92] Wolf SA, Boddeke HWGM, Kettenmann H (2017) Microglia in physiology and disease. Annu Rev Physiol 79:619–643. 10.1146/annurev-physiol-022516-034406 27959620

[CR93] Woodburn SC, Bollinger JL, Wohleb ES (2021) Synaptic and behavioral effects of chronic stress are linked to dynamic and sex-specific changes in microglia function and astrocyte dystrophy. Neurobiol Stress 14:100312. 10.1016/j.ynstr.2021.100312 PMC7970222 33748354

[CR94] Yamaguchi T, Danjo T, Pastan I, Hikida T, Nakanishi S (2013) Distinct roles of segregated transmission of the septo-habenular pathway in anxiety and fear. Neuron 78(3):537–544. 10.1016/j.neuron.2013.02.03523602500 PMC3654012

[CR95] Yeh L-F, Zuo S, Liu P-W (2024) Molecular diversity and functional dynamics in the central amygdala. Front Mol Neurosci 17:1364268. 10.3389/fnmol.2024.136426838419794 PMC10899328

[CR96] Yoo H, Kim HJ, Yang SH, Son GH, Gim JA, Lee HV, Kim H (2022) Gene expression profiling of the habenula in rats exposed to chronic restraint stress. Mol Cells 45(4):306–316. 10.14348/molcells.2022.225735534192 PMC9095505

[CR97] Zhao S, Umpierre AD, Wu LJ (2024) Tuning neural circuits and behaviors by microglia in the adult brain. Trends Neurosci 47(3):181–194. 10.1016/j.tins.2023.12.00338245380 PMC10939815

[CR98] Ziebell JM, Ray-Jones H, Lifshitz J (2017) Nogo presence is inversely associated with shifts in cortical microglial morphology following experimental diffuse brain injury. Neuroscience 359:209–223. 10.1016/j.neuroscience.2017.07.02728736137 PMC5597855

