# Supplementary material for: Differential effects of acute, chronic, and social defeat stress on microglial morphology in the basolateral and central amygdala, and the medial and lateral habenula
Source: Brain Struct Funct. 2026 Sep 4;231(8):133. doi: 10.1007/s00429-026-03172-y (PMC13545146; doi:10.1007/s00429-026-03172-y)
Supplement: Supplementary file 1 [file 429_2026_3172_MOESM1_ESM.pdf]

SUPPLEMENTAL FIGURES

Nieto-Quero A., Munoz-Martin J. et al., 2026

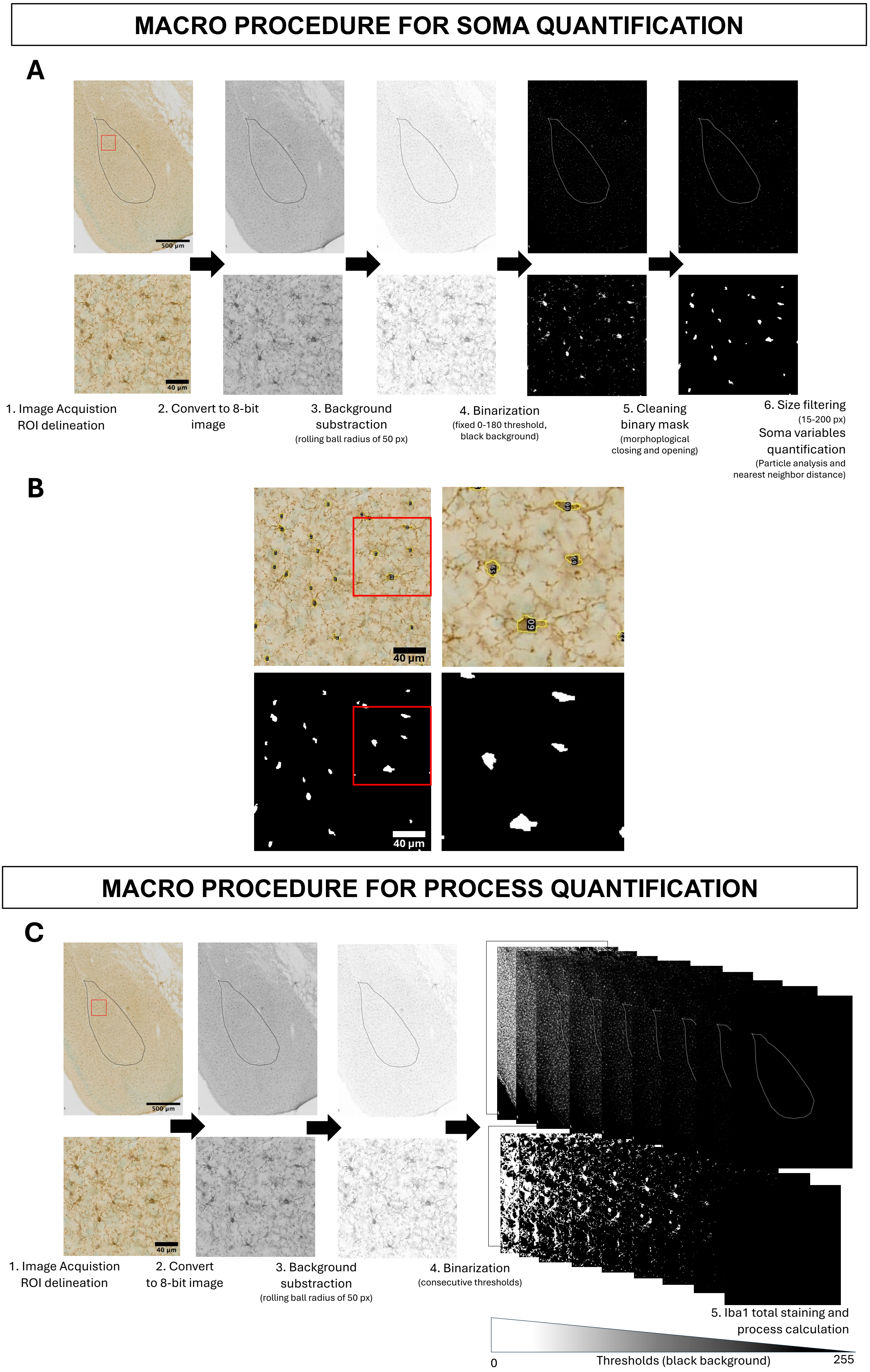

Fig S1. Visual procedures via macro automated analysis of microglial soma and processes in FIJI-ImageJ. (A) Representative images for each step of the macro used for soma quantification. As an example, the delineated Region of interest (ROI) represents the Basolateral Amygdala, adjusted using neuroanatomical references provided by Paxinos and Franklin 2004. Further downstream analysis was performed exclusively within the delineated ROIs. Red square indicates a magnified location, which is depicted in the image on the bottom where microglia cells can be observed. (B) Output images of the soma analysis in RGB and binary images. Each soma was labeled with a number and its morphological variables where obtained. Red squares indicate a magnified location, which is depicted in the images to the right. (C) Representative images for each step of the macro used for total Iba1 staining and process calculation. The percentage of total Iba1 staining was obtained for consecutive thresholds 0-255. For simplicity only ten of these thresholds where represented in the binary images.

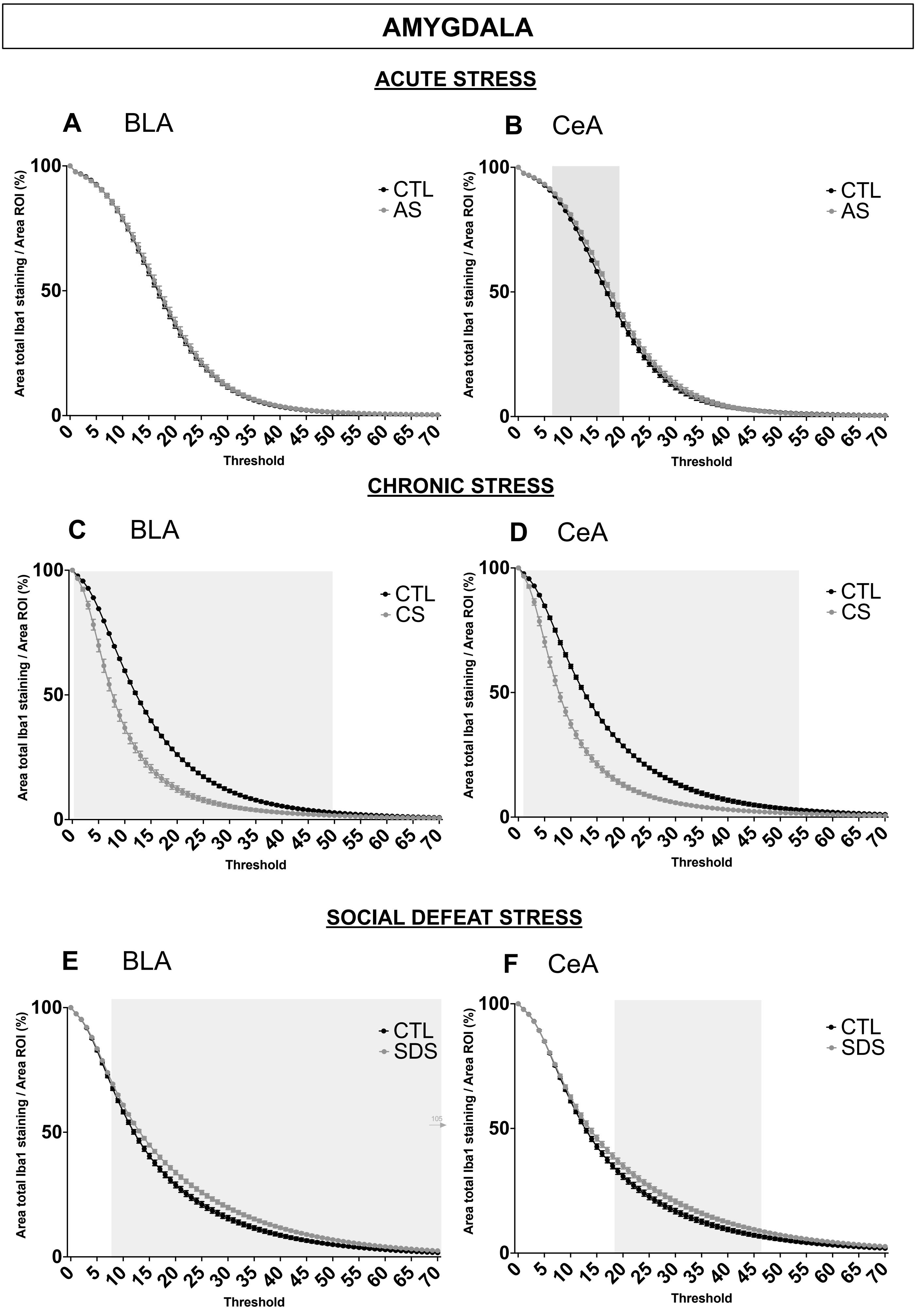

Fig S2. Total Iba1 staining in Amygdala subregions changes differently to diverse stress protocols. An exploratory analysis using an automated macro for process quantification was employed, obtaining the percentage of area of total Iba1 staining within different Regions of Interest (ROI). (A) Total Iba1 staining in Amygdala subregions after Acute Stress (AS), Basolateral Amygdala (BLA): no significant differences; measured as the percentage Area of total Iba1 staining normalized by the Area of the Region of Interest (ROI) along every grayscale threshold 0-255. For simplicity, only 0-70 grayscale thresholds were represented. (B) Central Amygdala (CeA): significant differences in the grayscale threshold range 7-19 as depicted by the gray rectangle. Chronic stress (CS); (C) BLA: significant threshold range 1-49 (D) CeA: significant threshold range 1-53. Social Defeat Stress (SDS); (E) BLA: significant threshold range 8-105 (F) CeA: significant threshold range 19-46. Data are presented as mean ± SEM and analyzed using Welch t-test. Gray rectangle depicts the range of significant points with a $p<0.05$. The mean threshold point of the significant range was used to obtain the values for calculation of the microglial processes. N=4 male mice per group per subregion.

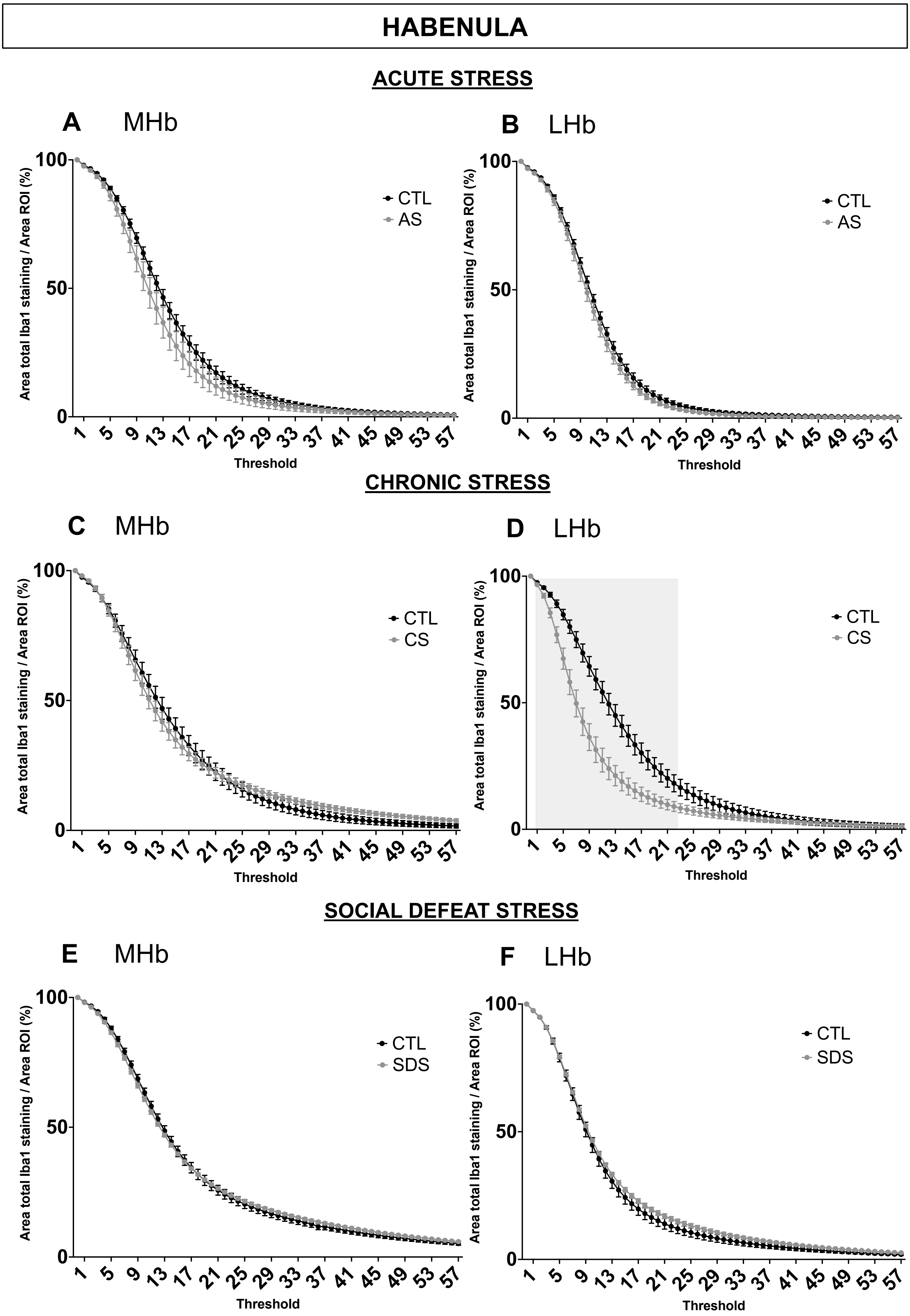

Fig S3. Total Iba1 staining in Habenula subregions changes exclusively under Chronic stress. An exploratory analysis using an automated macro for process quantification was employed, obtaining the percentage of area of total Iba1 staining within different Regions of Interest (ROI). (A) Total Iba1 staining in Habenula subregions after Acute Stress (AS), Medial Habenula (MHb): no significant differences; measured as the percentage Area of total Iba1 staining normalized by the Area of the Region of Interest (ROI) along every grayscale threshold 0-255. For simplicity, only 0-57 grayscale thresholds were represented. (B) Lateral Habenula (LHb): no significant differences. Chronic stress (CS); (C) MHb: no significant differences, (D) LHb: significant differences in the grayscale threshold range 1-22 as depicted by the gray rectangle. Social Defeat Stress (SDS); (E) MHb: no significant differences, (F) LHb: no significant differences. Data are presented as mean ± SEM and analyzed using Welch t-test. Gray rectangle depicts the range of significant thresholds with a $p<0.05$. N=4 male mice per group per subregion.

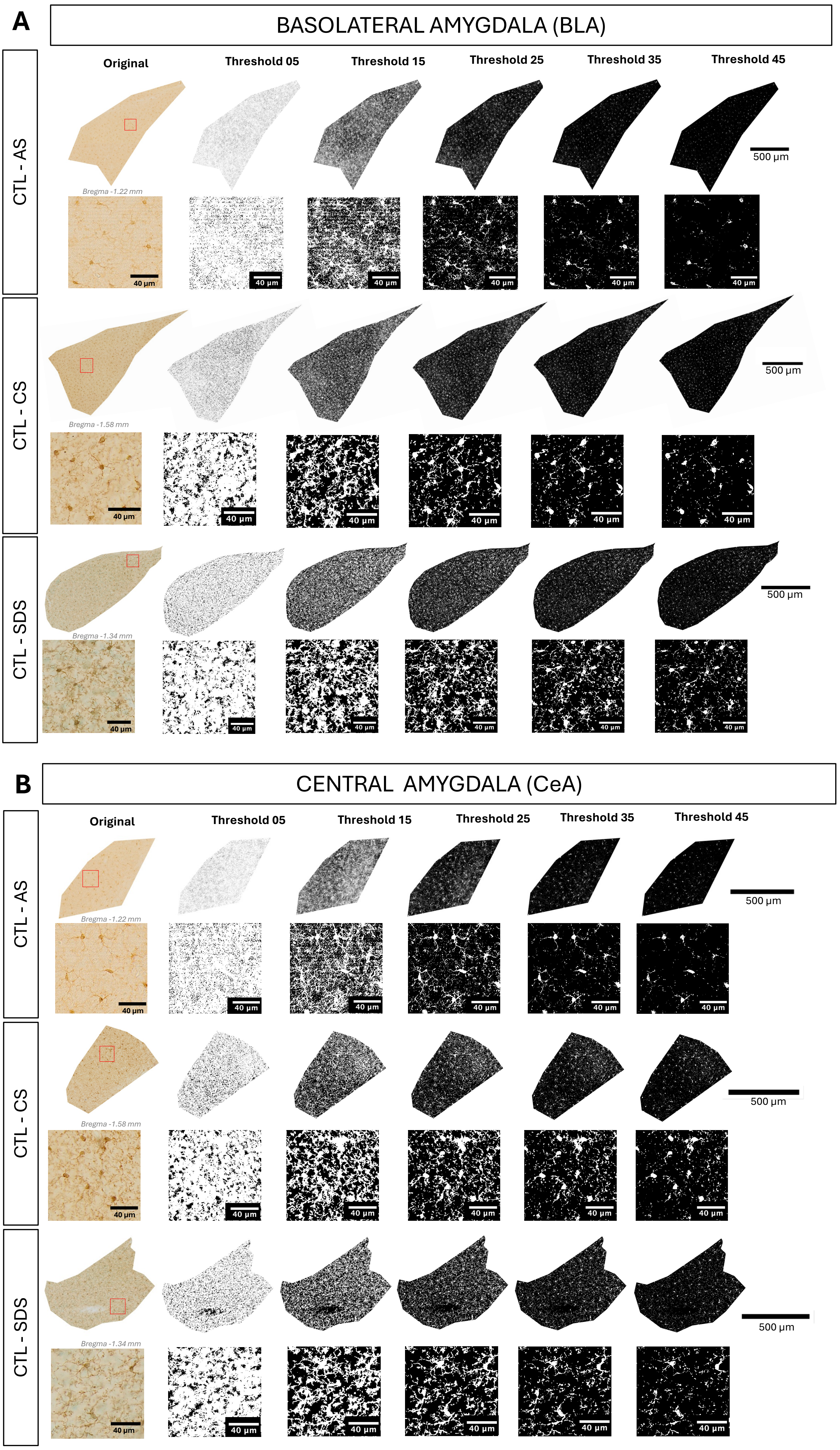

A
BASOLATERAL AMYGDALA (BLA)
Original
Threshold 05
Threshold 15
Threshold 25
Threshold 35
Threshold 45
CTL - AS
Bregma -1.22 mm
CTL - CS
Bregma -1.58 mm
CTL - SDS
Bregma -1.34 mm
500 µm
40 µm
B
CENTRAL AMYGDALA (CeA)
Original
Threshold 05
Threshold 15
Threshold 25
Threshold 35
Threshold 45
CTL - AS
Bregma -1.22 mm
CTL - CS
Bregma -1.58 mm
CTL - SDS
Bregma -1.34 mm
500 µm
40 µm

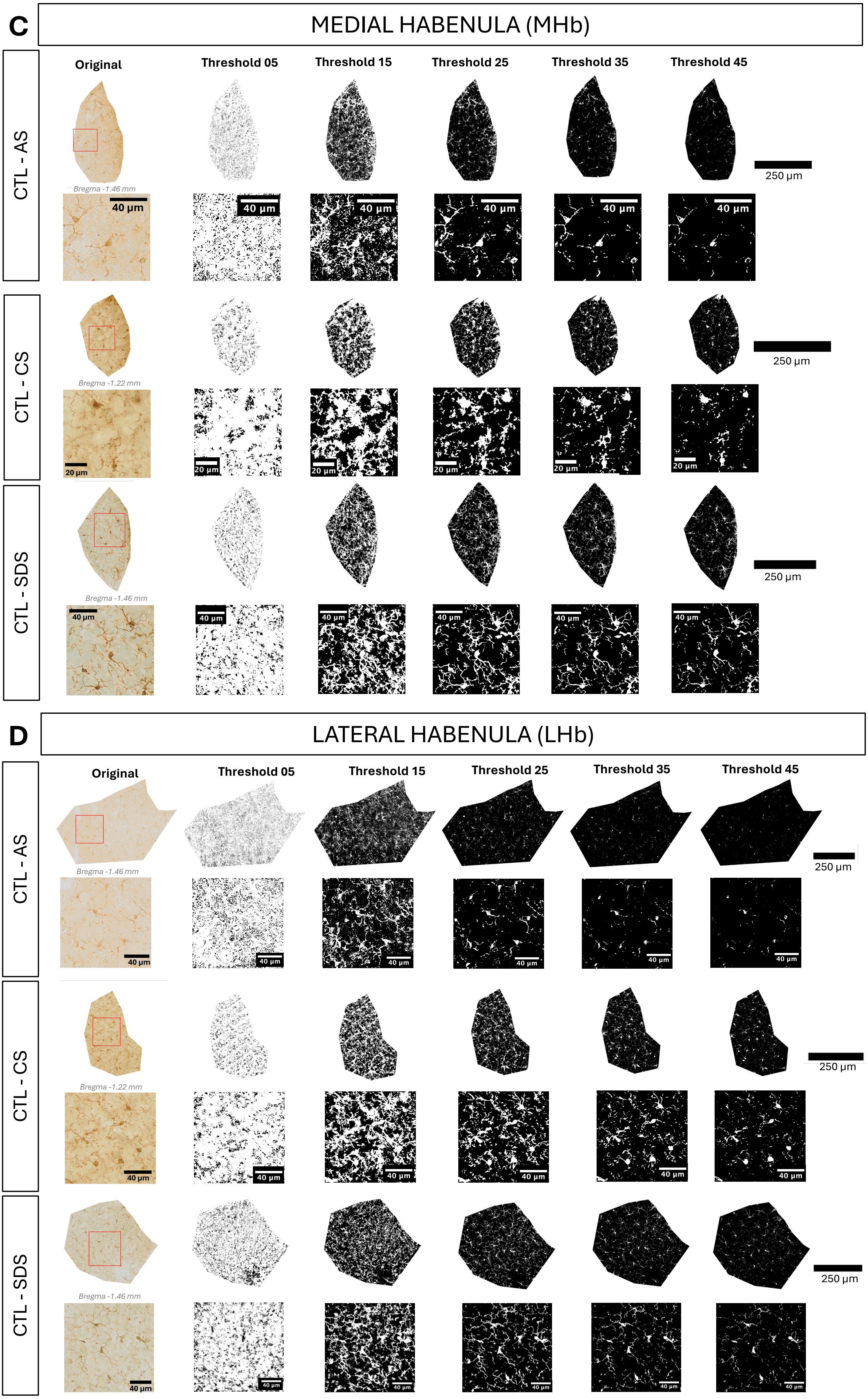

Fig S4. Microglial Iba1 staining and binary images for control groups in Amygdala and Habenula subregions throughout different grayscale thresholds as a sensitivity method to microglial process visualization. (A-B) Amygdala subregions for every control group in the different stress paradigms. AS: Acute stress, CS: Chronic Stress and SDS: Social Defeat Stress. ROI subregions were delineated using neuroanatomical references provided by Paxinos and Franklin 2004 and represented (top), magnification of microglia cells within the ROI is depicted (bottom) as indicated by the red square. Black and white binary images were modelled in FIJI/ImageJ to different grayscale thresholds via macro, to elucidate the range where microglial processes are visually represented. The percentage of Iba1 staining (white color within the binary images), in the range of threshold 25 to 45, was normalized by ROI area, averaged and used to calculate microglial processes area. (C-D) Habenula subregions for every control group in the different stress paradigms.

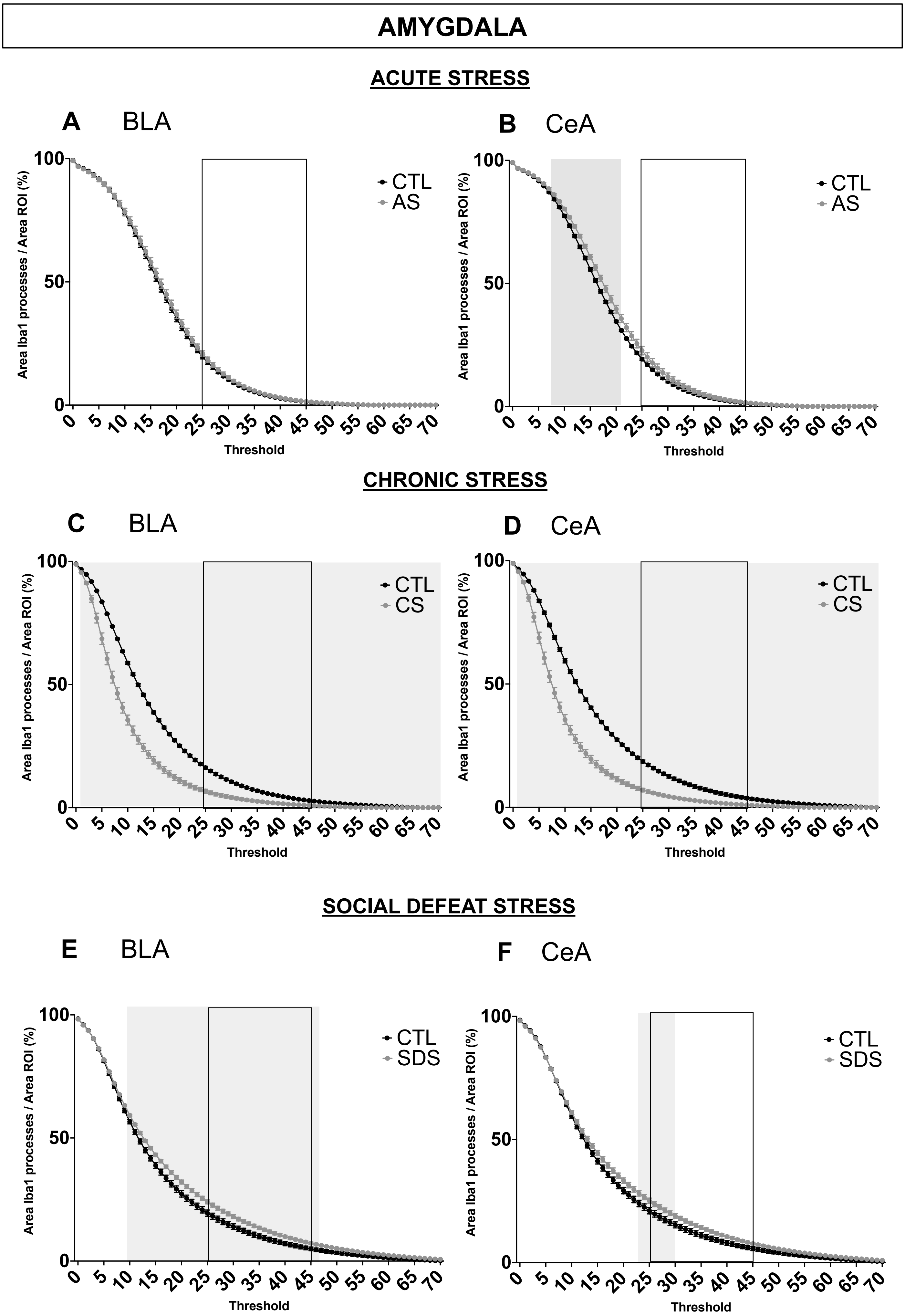

Fig S5. Iba1 staining associated to microglial processes in Amygdala subregions changes differently to diverse stress protocols. The percentage of staining associated to somas, derived from soma quantification analysis, was mathematically substracted to each threshold point in the total Iba1 staining curves, thus obtaining the Iba1 staining associated exclusively to microglial processes. (A) Iba1 processes staining in Amygdala subregions after Acute Stress (AS), Basolateral Amygdala (BLA): no significant differences; measured as the percentage Area of Iba1 staining associated to processes normalized by the Area of the Region of Interest (ROI) along every grayscale threshold 0-255. For simplicity, only 0-70 grayscale thresholds were represented. (B) Central Amygdala (CeA): significant differences in the grayscale threshold range 8-20 as depicted by the gray rectangle. Chronic stress (CS); (C) BLA: significant threshold range 1-70 (D) CeA: significant threshold range 1-70. Social Defeat Stress (SDS); (E) BLA: significant threshold range 10-47 (F) CeA: significant threshold range 23-29. Data are presented as mean ± SEM and analyzed using Welch t-test. Gray rectangle depicts the range of significant thresholds with a $p<0.05$. Black-outlined rectangle indicates the subset of thresholds where microglial Iba1 processes were visualized and averaged to obtain the percentage of Iba1 staining associated to processes. N=4 male mice per group per subregion.

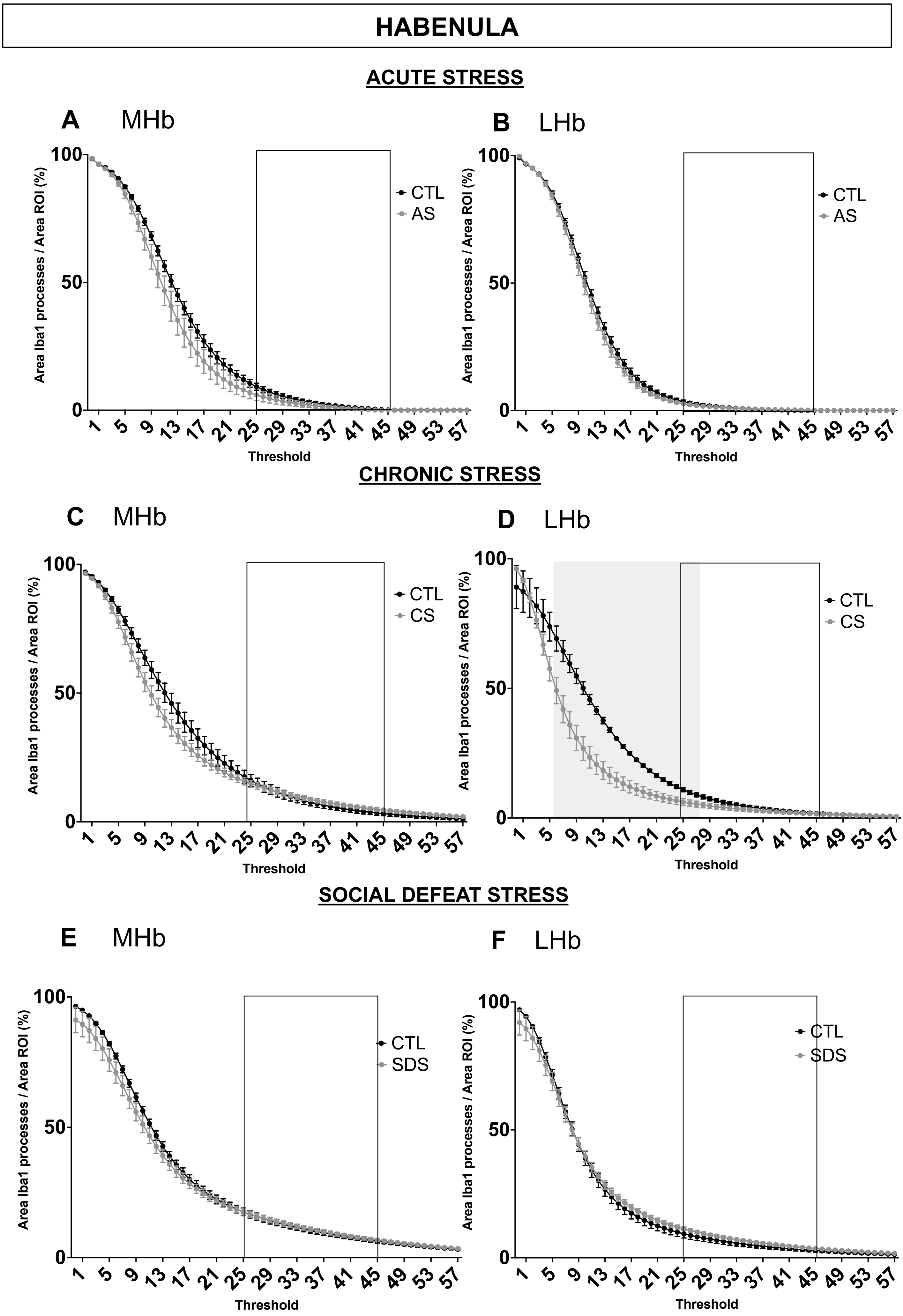

Fig S6. Iba1 staining associated to microglial processes in Habenula subregions changes mildly and exclusively under Chronic stress in a subregion-specific manner. The percentage of staining associated to somas, derived from soma quantification analysis, was mathematically substracted to each threshold point in the total Iba1 staining curves, thus obtaining the Iba1 staining associated exclusively to microglial processes. (A) Iba1 processes staining in Habenula subregions after Acute Stress (AS), Medial Habenula (MHb): no significant differences; measured as the percentage Area of Iba1 staining associated to processes normalized by the Area of the Region of Interest (ROI) along every grayscale threshold 0-255. For simplicity, only 0-57 grayscale thresholds were represented. (B) Lateral Habenula (LHb): no significant differences. Chronic stress (CS); (C) MHb: no significant differences, (D) LHb: significant differences in the grayscale threshold range 6-27 as depicted by the gray rectangle. Social Defeat Stress (SDS); (E) MHb: no significant differences, (F) LHb: no significant differences. Data are represented as mean ± SEM and analyzed using Welch t-test. Gray rectangle depicts the range of significant thresholds with a $p<0.05$. Black-outlined rectangle indicates the subset of thresholds where microglial Iba1 processes were visualized and averaged to obtain the percentage of Iba1 staining associated to processes. N=4 male mice per group per subregion.
