# Supplementary material for: Differential effects of acute, chronic, and social defeat stress on microglial morphology in the basolateral and central amygdala, and the medial and lateral habenula
Source: Brain Struct Funct. 2026 Sep 4;231(8):133. doi: 10.1007/s00429-026-03172-y (PMC13545146; doi:10.1007/s00429-026-03172-y)
Supplement: Supplementary file 2 [file 429_2026_3172_MOESM2_ESM.pdf]

```
/*
 * Macro realizada por: Manuela Vega Sánchez
 * Servicio Central de Informática (UMA)
 *
 *
 *
 */

input = getDirectory("Carpeta imágenes");
output = getDirectory("Carpeta resultados");
suffix = ".tif";

processFolder(input);

function processFolder(input) {
	list = getFileList(input);
	for (i = 0; i < list.length; i++) {
		if(File.isDirectory(input + list[i]))
			processFolder("" + input + list[i]);
		if(endsWith(list[i], suffix))
			processFile(input, output, list[i]);
	}
}

function processFile(input, output, file) {

		open(input + file);

		run("Set Scale...", "distance=1 known=0.345 pixel=1.000 unit=micron 
global");
		run("Set Measurements...", "area centroid perimeter shape feret's 
display redirect=None decimal=3");

		run("Duplicate...", "title=original");

		selectWindow(file);
		run("Split Channels");

		selectWindow(file + " (green)");
		close();
		selectWindow(file +" (red)");
		close();
		selectWindow(file +" (blue)");

		run("Duplicate...", " ");
//		run("8-bit");

		run("Subtract Background...", "rolling=50 light");
```

```
		setThreshold(0, 180);
//		waitForUser("selecciona");
		setOption("BlackBackground", true);
		run("Convert to Mask");
		run("Options...", "iterations=1 count=1 black do=Close");
		run("Options...", "iterations=2 count=1 black do=Open");

		waitForUser("Seleccionar zona1");
// Medir zona1
		run("Measure");
		saveAs("Results", output + file+ "Area1.txt");
		saveAs("Tiff", output + "BIN1" + file);

		run("Analyze Particles...", "size=15-200 display clear add in_situ");
		saveAs("Results", output + file+ "Somas1.txt");
		run("Nnd ");
		saveAs("Results", output + file+ "Distancias1.txt");


		selectWindow("original");
		roiManager("Show All");
		run("Flatten");

		saveAs("Jpeg", output + "Z1" + file);
		selectWindow("ROI Manager");
	run("Close");

		waitForUser("Seleccionar zona2");
// Medir zona2
		run("Measure");
		saveAs("Results", output + file+ "Area2.txt");
		saveAs("Tiff", output + "BIN2" + file);

		run("Analyze Particles...", "size=15-200 display clear add in_situ");
		saveAs("Results", output + file+ "Somas2.txt");
		run("Nnd ");
		saveAs("Results", output + file+ "Distancias2.txt");


		selectWindow("original");
		roiManager("Show All");
		run("Flatten");

		saveAs("Jpeg", output + "Z2" + file);
		selectWindow("ROI Manager");
	run("Close");

		run("Close All");
}
```
