# Supplementary material for: Differential effects of acute, chronic, and social defeat stress on microglial morphology in the basolateral and central amygdala, and the medial and lateral habenula
Source: Brain Struct Funct. 2026 Sep 4;231(8):133. doi: 10.1007/s00429-026-03172-y (PMC13545146; doi:10.1007/s00429-026-03172-y)
Supplement: Supplementary file 3 [file 429_2026_3172_MOESM3_ESM.docx]

**Table S1. Cluster validation and statistical analysis of microglial morphological classifications across experimental conditions.**
For each brain region (BLA, CeA, MHb, LHb) and stress condition (Acute Stress, Chronic Stress, Social Defeat Stress), clustering quality was evaluated using the Silhouette index, Calinski–Harabasz index, and Davies–Bouldin index. Statistical comparisons between clusters were performed for both soma area and processes parameters using *t-tests* or *Welch’s t-tests,* as appropriate and *p*-values. Effect sizes are provided as Hedges’s g. Statistically significant results (*p* < 0.05) are highlighted for each condition.

| **Region** | **Treatment** | **Silhouette Index** | **Calinski-Harabasz Index** | **Davies-Bouldin Index** | **Between SS/Total SS** | **Statistical Test (p-value). T-student o Welch. External validation** | **Effect Size**  **(Hedges)** |
| --- | --- | --- | --- | --- | --- | --- | --- |
| BLA | Acute Stress | 0.43 | 56.72 | 0.93 | 0.49 | Circularity: t_18.86_ = -1.51, p = 0.1470; Density; t_58_ = 3.09; p= 0.0030 | Circularity: *g*=-0.58, Density: *g*=0.84 |
|  | Chronic Stress | 0.55 | 49.76 | 0.84 | 0.57 | Circularity: t_26_= -4.10, p = 0.0004; Density; t_37_ =-0.87; p= 0.3909 | Circularity: *g*= -1.26, Density: *g*=0.27 |
|  | SDS | 0.44 | 55.47 | 0.98 | 0.49 | Circularity: t_57_= -2.23, p = 0.029; Density; t_22.36_ = 0.09; p= 0.92 | Circularity: *g*=-0.59, Density: *g*=0.03 |
| CeA | Acute Stress | 0.41 | 44.40 | 1.03 | 0.48 | Circularity: t_40.4_= 3.33, p = 0.001851866; Density; t_58_ = -4.88; p =0.000011 | Circularity: *g*=0.83, Density: *g*=-1.37 |
|  | Chronic Stress | 0.50 | 33.04 | 0.98 | 0.51 | Circularity: t_32_= -2.03, p = 0.051; Density; t_32_ = -2.06; p=0.04 | Circularity: *g*=-0.68, Density: *g*=-0.692 |
|  | SDS | 0.44 | 48.55 | 1.00 | 0.49 | Circularity: t_51_= 4.48, p <0.0001; Density; t_24.68_ = -0.40; p=0.69 | Circularity: *g*=1.24, Density: *g*=-0.12 |
| MHb | Acute Stress | 0.41 | 19.77 | 0.92 | 0.46 | Circularity: t_23_= -2.85, p = 0.009; Density; t_23_ = 4.88; p<0.001 | Circularity: *g*=-1.14, Density: *g*=1.96 |
|  | Chronic Stress | 0.37 | 19.03 | 1.09 | 0.44 | Circularity: t_24_= -2.522, p = 0.018; Density; t_24_ = 2.048; p=0.051 | Circularity: *g*=-0.96, Density: *g*=0.80 |
|  | SDS | 0.36 | 19.57 | 1.21 | 0.36 | Circularity: t_35_= -2.381, p = 0.02; Density; t_35_ = 1.512; p=0.139 | Circularity: *g*=- 0.74, Density: *g*=0.49 |
| LHb | Acute Stress | 0.65 | 21.58 | 0.93 | 0.48 | Circularity: t_23_= 2.57, p = 0.017; Density; t_23_ = -0.70; p=0.486 | Circularity: *g*=1.52, Density: *g*=-0.42 |
|  | Chronic Stress | 0.55 | 25.65 | 0.94 | 0.53 | Circularity: t_23_= -2.60, p = 0.016; Density; t_58_ = 4.969; p=0.001 | Circularity: *g*=-1.12, Density: *g*=2.14 |
|  | SDS | 0.43 | 36.69 | 0.88 | 0.51 | Circularity: t_13.02_ = -3.20, p = 0.007; Density; t_34_ = 3.90; p= 0.004 | Circularity: *g*=-1.38, Density: *g*=1.38 |

*Internal validation indices are reported for each exploratory K = 2 cluster solution. Cluster solutions with Silhouette values below 0.50 were interpreted descriptively and were not considered to indicate robust cluster separation. Cluster assignments should therefore be interpreted as descriptive morphometric profiles rather than discrete biological states.*

**Table S2. Animal-level bootstrap stability analysis of exploratory k-means clustering solutions.**

| **Paradigm** | **Region** | **n animals** | **n sections** | **Bootstrap iterations (successful/total)** | **ARI, mean ± SD** | **Mean Jaccard, mean ± SD** | **Cluster 1 Jaccard, mean ± SD** | **Cluster 2 Jaccard, mean ± SD** | **Bootstrap Stability interpretation** |
| --- | --- | --- | --- | --- | --- | --- | --- | --- | --- |
| Acute stress | BLA | 8 | 60 | 100/100 | 0.874 ± 0.205 | 0.932 ± 0.108 | 0.947 ± 0.096 | 0.917 ± 0.122 | Very high stability |
| Acute stress | CeA | 8 | 50 | 100/100 | 0.729 ± 0.208 | 0.860 ± 0.113 | 0.876 ± 0.117 | 0.844 ± 0.111 | Generally high stability, with variability across bootstrap samples |
| Acute stress | MHb | 8 | 25 | 100/100 | 0.697 ± 0.319 | 0.836± 0.184 | 0.798 ± 0.233 | 0.874± 0.129 | Acceptable stability |
| Acute stress | LHb | 8 | 25 | 100/100 | 0.695 ± 0.292 | 0.802 ± 0.185 | 0.941 ± 0.084 | 0.663 ± 0.305 | Acceptable but uneven stability |
| Chronic stress | BLA | 8 | 39 | 100/100 | 0.920 ± 0.203 | 0.958 ± 0.111 | 0.960 ± 0.107 | 0.956 ± 0.116 | High stability |
| Chronic stress | CeA | 8 | 34 | 100/100 | 0.799 ± 0.327 | 0.894 ± 0.178 | 0.900 ± 0.169 | 0.888 ± 0.189 | High average stability, with variability across bootstrap samples |
| Chronic stress | MHb | 8 | 26 | 100/100 | 0.589 ± 0.303 | 0.787 ± 0.154 | 0.831 ± 0.132 | 0.742 ± 0.181 | Moderate/acceptable but uneven stability |
| Chronic stress | LHb | 8 | 25 | 100/100 | 0.793 ± 0.227 | 0.877 ± 0.135 | 0.935 ± 0.073 | 0.819 ± 0.200 | High average but uneven stability |
| SDS | BLA | 8 | 59 | 100/100 | 0.875 ± 0.200 | 0.935 ± 0.106 | 0.926 ± 0.110 | 0.943 ± 0.103 | Very high stability |
| SDS | CeA | 8 | 53 | 100/100 | 0.903 ± 0.188 | 0.949 ± 0.102 | 0.946 ± 0.098 | 0.953 ± 0.107 | Very high stability |
| SDS | MHb | 8 | 37 | 100/100 | 0.358 ± 0.433 | 0.660 ± 0.231 | 0.664 ± 0.241 | 0.657 ± 0.230 | Moderate stability |
| SDS | LHb | 8 | 36 | 100/100 | 0.672 ± 0.384 | 0.823 ± 0.207 | 0.803 ± 0.221 | 0.843 ± 0.195 | Acceptable but variable stability |

**Note.** Summary of bootstrap stability metrics across stress paradigms and brain regions. Values are reported as mean ± SD across 100 animal-level bootstrap iterations.

ARI, Adjusted Rand Index. Jaccard values indicate the similarity between the original k-means clustering solution and bootstrap-derived clustering solutions projected onto the original dataset. In each bootstrap iteration, animals were sampled with replacement, and all sections belonging to each selected animal were retained. K-means clustering was repeated using the same settings as in the original analysis (k = 2, nstart = 10). These analyses assess the stability of exploratory morphometric clustering solutions under animal-level resampling and do not constitute independent molecular, functional, or behavioral validation of discrete microglial states. Therefore, all cluster-related findings should be interpreted descriptively.

**Table S3. Animal-level cluster membership by stress paradigm, region, and treatment group**

| **Stress paradigm** | **Region** | **Animal** | **Group / treatment** | **N sections** | **N cluster 1** | **N cluster 2** | **% cluster 1** | **% cluster 2** |
| --- | --- | --- | --- | --- | --- | --- | --- | --- |
| Acute stress | BLA | ANIMAL 1 | Control | 8 | 5 | 3 | 62 | 38 |
| Acute stress | BLA | ANIMAL 6 | Control | 8 | 6 | 2 | 75 | 25 |
| Acute stress | BLA | ANIMAL 9 | Control | 5 | 3 | 2 | 60 | 40 |
| Acute stress | BLA | ANIMAL 14 | Control | 8 | 7 | 1 | 88 | 12 |
| Acute stress | BLA | ANIMAL 2 | Acute stress | 7 | 4 | 3 | 57 | 43 |
| Acute stress | BLA | ANIMAL 5 | Acute stress | 6 | 6 | 0 | 100 | 0 |
| Acute stress | BLA | ANIMAL 10 | Acute stress | 9 | 4 | 5 | 44 | 56 |
| Acute stress | BLA | ANIMAL 13 | Acute stress | 9 | 6 | 3 | 67 | 33 |
| Acute stress | CeA | ANIMAL 1 | Control | 7 | 1 | 6 | 14 | 85 |
| Acute stress | CeA | ANIMAL 6 | Control | 6 | 5 | 1 | 83 | 17 |
| Acute stress | CeA | ANIMAL 9 | Control | 4 | 3 | 1 | 75 | 25 |
| Acute stress | CeA | ANIMAL 14 | Control | 8 | 8 | 0 | 100 | 0 |
| Acute stress | CeA | ANIMAL 2 | Acute stress | 5 | 0 | 5 | 0 | 100 |
| Acute stress | CeA | ANIMAL 5 | Acute stress | 5 | 1 | 4 | 20 | 80 |
| Acute stress | CeA | ANIMAL 10 | Acute stress | 8 | 4 | 4 | 50 | 50 |
| Acute stress | CeA | ANIMAL 13 | Acute stress | 7 | 7 | 0 | 100 | 0 |
| Acute stress | MHb | ANIMAL 1 | Control | 4 | 3 | 1 | 75 | 25 |
| Acute stress | MHb | ANIMAL 6 | Control | 3 | 1 | 2 | 33 | 67 |
| Acute stress | MHb | ANIMAL 9 | Control | 3 | 0 | 3 | 0 | 100 |
| Acute stress | MHb | ANIMAL 14 | Control | 2 | 2 | 0 | 100 | 0 |
| Acute stress | MHb | ANIMAL 2 | Acute stress | 5 | 3 | 2 | 60 | 40 |
| Acute stress | MHb | ANIMAL 5 | Acute stress | 2 | 2 | 0 | 100 | 0 |
| Acute stress | MHb | ANIMAL 10 | Acute stress | 3 | 0 | 3 | 0 | 100 |
| Acute stress | MHb | ANIMAL 13 | Acute stress | 3 | 3 | 0 | 100 | 0 |
| Acute stress | LHb | ANIMAL 1 | Control | 4 | 4 | 0 | 100 | 0 |
| Acute stress | LHb | ANIMAL 14 | Control | 2 | 1 | 1 | 50 | 50 |
| Acute stress | LHb | ANIMAL 6 | Control | 3 | 3 | 0 | 100 | 0 |
| Acute stress | LHb | ANIMAL 9 | Control | 3 | 2 | 1 | 67 | 33 |
| Acute stress | LHb | ANIMAL 10 | Acute stress | 3 | 3 | 0 | 100 | 0 |
| Acute stress | LHb | ANIMAL 13 | Acute stress | 3 | 3 | 0 | 100 | 0 |
| Acute stress | LHb | ANIMAL 2 | Acute stress | 5 | 4 | 1 | 80 | 20 |
| Acute stress | LHb | ANIMAL 5 | Acute stress | 2 | 2 | 0 | 100 | 0 |
| Chronic stress | BLA | ANIMAL 81 | Control | 6 | 6 | 0 | 100 | 0 |
| Chronic stress | BLA | ANIMAL 82 | Control | 5 | 5 | 0 | 100 | 0 |
| Chronic stress | BLA | ANIMAL 84 | Control | 4 | 4 | 0 | 100 | 0 |
| Chronic stress | BLA | ANIMAL 85 | Control | 4 | 4 | 0 | 100 | 0 |
| Chronic stress | BLA | ANIMAL 1 | Chronic stress | 0 | 5 | 5 | 0 | 100 |
| Chronic stress | BLA | ANIMAL 15 | Chronic stress | 0 | 6 | 6 | 0 | 100 |
| Chronic stress | BLA | ANIMAL 16 | Chronic stress | 0 | 3 | 3 | 0 | 100 |
| Chronic stress | BLA | ANIMAL 6 | Chronic stress | 0 | 6 | 6 | 0 | 100 |
| Chronic stress | CeA | ANIMAL 81 | Control | 6 | 6 | 0 | 100 | 0 |
| Chronic stress | CeA | ANIMAL 82 | Control | 5 | 5 | 0 | 100 | 0 |
| Chronic stress | CeA | ANIMAL 84 | Control | 4 | 4 | 0 | 100 | 0 |
| Chronic stress | CeA | ANIMAL 85 | Control | 4 | 4 | 0 | 100 | 0 |
| Chronic stress | CeA | ANIMAL 1 | Chronic stress | 4 | 0 | 4 | 0 | 100 |
| Chronic stress | CeA | ANIMAL 15 | Chronic stress | 6 | 0 | 6 | 0 | 100 |
| Chronic stress | CeA | ANIMAL 16 | Chronic stress | 2 | 0 | 2 | 0 | 100 |
| Chronic stress | CeA | ANIMAL 6 | Chronic stress | 4 | 0 | 4 | 0 | 100 |
| Chronic stress | MHb | ANIMAL 81 | Control | 2 | 1 | 1 | 50 | 50 |
| Chronic stress | MHb | ANIMAL 82 | Control | 3 | 0 | 3 | 0 | 100 |
| Chronic stress | MHb | ANIMAL 84 | Control | 2 | 2 | 0 | 100 | 0 |
| Chronic stress | MHb | ANIMAL 85 | Control | 1 | 1 | 0 | 100 | 0 |
| Chronic stress | MHb | ANIMAL 1 | Chronic stress | 4 | 4 | 0 | 100 | 0 |
| Chronic stress | MHb | ANIMAL 15 | Chronic stress | 5 | 4 | 1 | 80 | 20 |
| Chronic stress | MHb | ANIMAL 16 | Chronic stress | 5 | 3 | 2 | 60 | 40 |
| Chronic stress | MHb | ANIMAL 6 | Chronic stress | 4 | 1 | 3 | 25 | 75 |
| Chronic stress | LHb | ANIMAL 81 | Control | 2 | 2 | 0 | 100 | 0 |
| Chronic stress | LHb | ANIMAL 82 | Control | 3 | 1 | 2 | 33 | 67 |
| Chronic stress | LHb | ANIMAL 84 | Control | 2 | 2 | 0 | 100 | 0 |
| Chronic stress | LHb | ANIMAL 85 | Control | 1 | 1 | 0 | 100 | 0 |
| Chronic stress | LHb | ANIMAL 1 | Chronic stress | 4 | 3 | 1 | 75 | 25 |
| Chronic stress | LHb | ANIMAL 15 | Chronic stress | 5 | 5 | 0 | 100 | 0 |
| Chronic stress | LHb | ANIMAL 16 | Chronic stress | 5 | 3 | 2 | 60 | 40 |
| Chronic stress | LHb | ANIMAL 6 | Chronic stress | 4 | 2 | 2 | 50 | 50 |
| SDS | BLA | ANIMAL 9 | Control | 7 | 7 | 0 | 100 | 0 |
| SDS | BLA | ANIMAL 10 | Control | 7 | 5 | 2 | 71 | 29 |
| SDS | BLA | ANIMAL 11 | Control | 6 | 2 | 4 | 33 | 67 |
| SDS | BLA | ANIMAL 12 | Control | 8 | 3 | 5 | 37 | 63 |
| SDS | BLA | ANIMAL 2 | Stress (SDS) | 7 | 1 | 6 | 14 | 86 |
| SDS | BLA | ANIMAL 3 | Stress (SDS) | 8 | 0 | 8 | 0 | 100 |
| SDS | BLA | ANIMAL 4 | Stress (SDS) | 8 | 1 | 7 | 12 | 88 |
| SDS | BLA | ANIMAL 5 | Stress (SDS) | 8 | 3 | 5 | 37 | 63 |
| SDS | CeA | ANIMAL 9 | Control | 7 | 7 | 0 | 100 | 0 |
| SDS | CeA | ANIMAL 10 | Control | 7 | 4 | 3 | 57 | 43 |
| SDS | CeA | ANIMAL 11 | Control | 6 | 2 | 4 | 33 | 67 |
| SDS | CeA | ANIMAL 12 | Control | 7 | 3 | 4 | 43 | 57 |
| SDS | CeA | ANIMAL 2 | Stress (SDS) | 6 | 2 | 4 | 33 | 67 |
| SDS | CeA | ANIMAL 3 | Stress (SDS) | 6 | 1 | 5 | 17 | 83 |
| SDS | CeA | ANIMAL 4 | Stress (SDS) | 8 | 1 | 7 | 12 | 88 |
| SDS | CeA | ANIMAL 5 | Stress (SDS) | 6 | 1 | 5 | 17 | 83 |
| SDS | MHb | ANIMAL 9 | Control | 5 | 4 | 1 | 80 | 20 |
| SDS | MHb | ANIMAL 10 | Control | 5 | 5 | 0 | 100 | 0 |
| SDS | MHb | ANIMAL 11 | Control | 3 | 1 | 2 | 33 | 67 |
| SDS | MHb | ANIMAL 12 | Control | 5 | 3 | 2 | 60 | 40 |
| SDS | MHb | ANIMAL 2 | Stress (SDS) | 5 | 4 | 1 | 80 | 20 |
| SDS | MHb | ANIMAL 3 | Stress (SDS) | 5 | 1 | 4 | 20 | 80 |
| SDS | MHb | ANIMAL 4 | Stress (SDS) | 5 | 1 | 4 | 20 | 80 |
| SDS | MHb | ANIMAL 5 | Stress (SDS) | 4 | 3 | 1 | 75 | 25 |
| SDS | LHb | ANIMAL 10 | Control | 5 | 5 | 0 | 100 | 0 |
| SDS | LHb | ANIMAL 11 | Control | 3 | 0 | 3 | 0 | 100 |
| SDS | LHb | ANIMAL 12 | Control | 5 | 2 | 3 | 40 | 60 |
| SDS | LHb | ANIMAL 9 | Control | 5 | 1 | 4 | 20 | 80 |
| SDS | LHb | ANIMAL 2 | Stress (SDS) | 5 | 2 | 3 | 40 | 60 |
| SDS | LHb | ANIMAL 3 | Stress (SDS) | 4 | 0 | 4 | 0 | 100 |
| SDS | LHb | ANIMAL 4 | Stress (SDS) | 5 | 0 | 5 | 0 | 100 |
| SDS | LHb | ANIMAL 5 | Stress (SDS) | 4 | 1 | 3 | 25 | 75 |
